# N-Substituted 2-(Benzenosulfonyl)-1-Carbotioamide Derivatives Exert Antimicrobial and Cytotoxic Effects via Aldehyde Dehydrogenase Pathway: Synthesis, In Silico and In Vitro Studies

**DOI:** 10.3390/ph16121706

**Published:** 2023-12-08

**Authors:** Lucja Walczak-Nowicka, Anna Biernasiuk, Wojciech Ziemichód, Zbigniew Karczmarzyk, Mateusz Kwaśnik, Paweł Kozyra, Waldemar Wysocki, Agnieszka Stenzel-Bembenek, Dorota Kowalczuk, Mariola Herbet, Monika Pitucha

**Affiliations:** 1 Chair and Department of Toxicology, Faculty of Pharmacy, Medical University of Lublin, Jaczewskiego 8b, 20-090 Lublin, Poland; lucja.walczak-nowicka@umlub.pl (L.W.-N.); mariola.herbet@umlub.pl (M.H.); 2 Department of Pharmaceutical Microbiology, Faculty of Pharmacy, Medical University of Lublin, Chodzki 1, 20-093 Lublin, Poland; anna.biernasiuk@umlub.pl; 3 Independent Radiopharmacy Unit, Faculty of Pharmacy, Medical University of Lublin, Chodzki 4a, 20-093 Lublin, Poland; wojciechziemichod@gmail.com (W.Z.); pawelkozyra@umlub.pl (P.K.); 4 Institute of Chemistry, University of Siedlce, 3 Maja 54, 08-110 Siedlce, Poland; zbigniew.karczmarzyk@uph.edu.pl (Z.K.);; 5 Department of Molecular Biology, Faculty of Medicine, The John Paul II Catholic University of Lublin, Konstantynów 1J/4.03, 20-708 Lublin, Poland; mateusz.kwasnik@kul.pl; 6 Department of Biochemistry and Molecular Biology, Faculty of Medical Sciences, Medical University of Lublin, Chodzki 1, 20-093 Lublin, Poland; 7 Department of Medicinal Chemistry, Faculty of Pharmacy, Medical University of Lublin, Jaczewskiego 4, 20-090 Lublin, Poland

**Keywords:** thiosemicarbazide, antimicrobial, anticancer: in silico, aldehyde dehydrogenase, docking

## Abstract

A series of N-Substituted 2-(benzenosulfonyl)-1-carbotioamide derivatives (WZ1–WZ4) were synthesized and characterized using spectral methods. A comprehensive activity study was performed for each compound. All compounds were tested for antibacterial activity. Moreover, in silico studies were carried out to determine the anticancer potential of the designed WZ1–WZ4 ligands. Based on molecular docking, aldehyde dehydrogenase was selected as a molecular target. The obtained data were compared with experimental data in vitro tests. Novel hybrids of the thiosemicarbazide scaffold and sulfonyl groups may have promising anticancer activity via the aldehyde dehydrogenase pathway. The best candidate for further studies appears to be WZ2, due to its superior selectivity in comparison to the other tested compounds.

## 1. Introduction

Sulfonamides and sulfanilamide derivatives constitute a large group of drugs with bacteriostatic properties and at the same time an acceptable level of toxicity towards higher organisms. Many broad-spectrum sulfonamide derivatives have been developed as chemotherapeutic and preventive agents for a variety of diseases [1,2]. Derivatives containing a sulfone moiety are constantly modified and tested as substances with anti-tuberculosis activity [3], anti-inflammatory and anticancer properties [4,5,6] and as alpha-deglucosidase inhibitors [7,8]. Moreover, it has been shown that sulfonyl derivatives of 5-fluorosubstituted benzimidazole have effective affinity for the MAO-B 2C65 responsible for Parkinson’s disease (PD) [9]. Sulfonamides are predominantly used in antibacterial therapies [10,11,12]. Their spectrum of action covers both Gram-positive bacteria such as *Staphylococcus* spp. or *Streptococcus* spp., but also Gram-negative bacteria.

On the other hand, thiosemicarbazide derivatives with anticonvulsant [13], antituberculosis [14] and antiviral activity [15] are widely described in the literature. The antibacterial activity of thiosemicarbazide derivatives against *S. aureus*, *S. epidermidis*, *S. mutans* and *S. sanguinis* is noteworthy, with moderate cytotoxicity and good therapeutic safety.

This property is particularly important from the point of view of the increasing drug resistance among both Gram-positive bacteria, such as those mentioned above, and also Gram-negative bacilli [16,17,18]. The number of drug-resistant bacteria is increasing year on year in all parts of the world. 

Their combination with sulfone moiety may prove beneficial in the context of overcoming drug resistance, due to the simultaneous beneficial effect of thiosemicarbazides and sulfonamides in terms of antimicrobial properties.

Currently, there are several chemotherapeutic agents on the pharmaceutical market but there is still an upward trend in the incidence and mortality of malignant tumors cells [19]. A significant problem in the treatment of cancer is that drugs on the market often fail to cure patients despite early detection [20]. In addition, patients experience several complications during chemotherapy and also during radiotherapy. These include gastrointestinal problems, nausea and vomiting, but also complications that pose a direct threat to the patient’s life: pancytopenias, secondary tumors and thromboembolic complications.

The search for the new compounds that could contribute to improving the curability of malignant tumors while minimizing side effects seems crucial and justified [21]. Due to the wide range of applications in medicinal and applied chemistry, thiosemicarbazides are also modified for anticancer activity [20,21,22]. Many studies have shown that thiosemicarbazides can combine various strategies used in cancer therapy, including effects on enzymes involved in carcinogenesis, and anti-angiogenic, antioxidant or anti-inflammatory properties [22,23,24]. However, a key feature is that they have been shown to possess antiproliferative properties in several studies [25,26,27,28,29]. Therefore, the thiosemicarbazide scaffold is responsible for promising biological properties. One study compared a semicarbazide molecule and a thiosemicarbazide bearing a 4-nitrophenyl substituent. The compound with the thiosemicarbazide scaffold possessed stronger antiproliferative properties [30]. Importantly, it is also believed that it is not the structure of the thiosemicarbazide itself that is important in the anticancer activity, but its structure as a whole [31]. Studies emphasize the importance of the nature and position of the radicals in the molecule.

Furthermore, it is believed that the presence of a thiosemicarbazide–sulfone bond may also be important for anti-tumor activity [4,5,6,32].

In our work, hybrid compounds bearing both benzenesulfone and thiosemicarbazide groups were designed and synthesized. In the present article we verified the antimicrobial activity of compounds WZ1–WZ4 against reference bacteria and fungi strains. We also examined their toxicity to erythrocytes. Finally, based on the virtual screening and molecular docking, aldehyde dehydrogenase inhibitors were indicated as a potential molecular target. The results obtained using in silico tests were experimentally verified using in vitro tests. 

## 2. Results

### 2.1. Chemistry

**Scheme 1:**
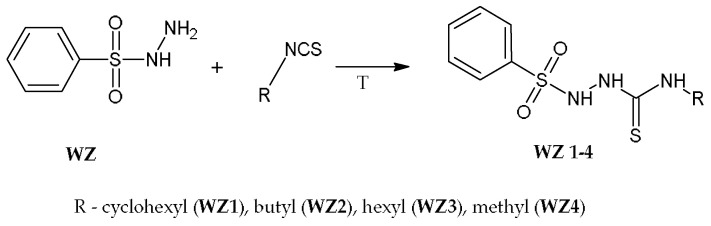
Synthesis path of new N-Substituted 2-(benzenosulfonyl)-1-carbotioamide derivatives.

In medicinal chemistry, the development of new, effective drugs containing nitrogen and sulfur heteroatoms is of great importance. A comprehensive and rational approach to drug design is molecular hybridization (MH), which involves combining two pharmacophore molecules with distinct intrinsic activities into a single scaffold to increase their therapeutic potential. Sulfonylo-thiosemicarbazide hybrids are a valuable scaffold, which is related to their utility in the treatment of many diseases and simple synthetic pathways. The title compounds were obtained by reacting benzenesulfonohydrazide WZ with cyclohexyl, butyl, hexyl and methyl isothiocyanate (Scheme 1). The new connections (compounds WZ1–WZ4) were characterized using ^1^H, ^13^C NMR and IR spectroscopic methods. All compounds were tested for antibacterial activity. In silico tests of the predisposition of WZ1–WZ4 compounds towards anticancer activity and ADMET analysis were performed. A potential molecular target was proposed on the basis of molecular docking.

### 2.2. X-ray Investigation

**Figure 1:**
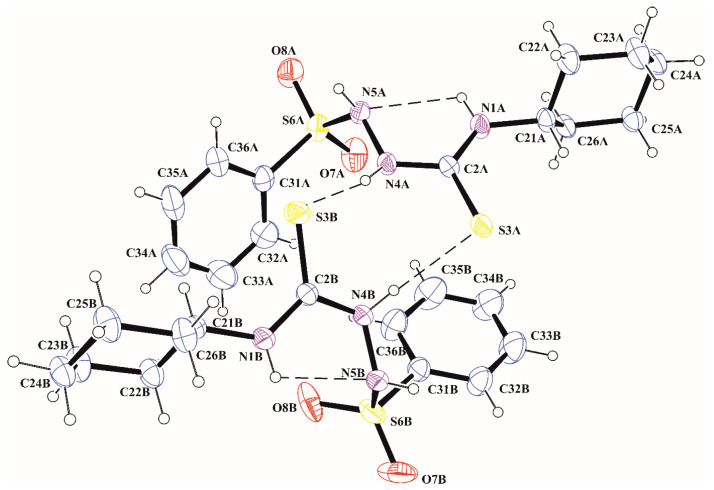
The molecules of WZ1 with atom labels and displacement ellipsoids (30% probability level). Dashed lines indicate intra- and intermolecular hydrogen bonds.

**Figure 2:**
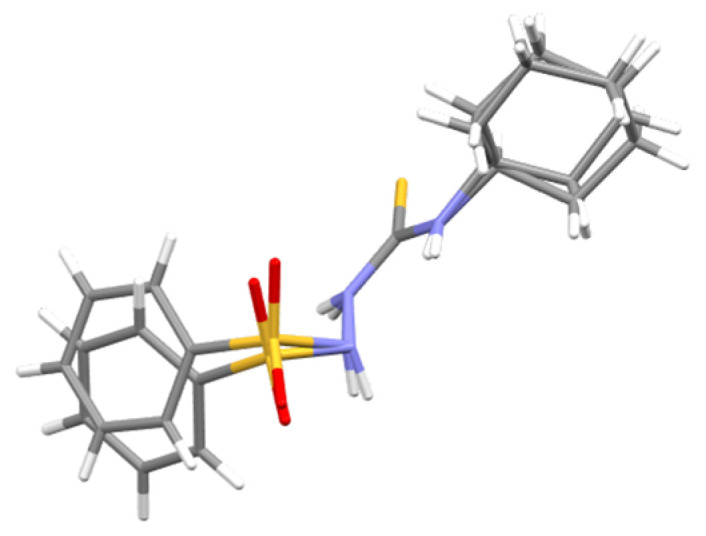
A overlay of molecules A and B by fitting of the atoms positions of the thiourea system (RMS = 0.0123 Å).

The X-ray investigation was performed for the model compound WZ1 in order to confirm the synthetic route, assumed molecular structure as well as to characterize the geometry, conformations and intra- and intermolecular interactions in the crystal in terms of potential biological activity. The X-ray analysis showed that the compound WZ1 crystalizes in *P*2_1_/*c* space group with two molecules in the asymmetric part of the unit cell. The X-ray structures of molecules A and B in the conformations observed in the crystalline state are presented in Figure 1. The two crystallographically independent molecules have very similar geometry, but slightly different conformations, as shown in the Figure 2 where the overlay of molecules A and B with the positions of non-hydrogen atoms in the thiourea planar system is presented.

The geometry of the biological active sulfonamide system in molecules A and B is typical for this type of system [33]. The S=O bond lengths are in the range from 1.417(3) to 1.427(2) Å and the S6—C31 and S6—N5 bond lengths are 1.754(4) and 1.660(3) Å in A and 1.745(3) and 1.657(3) Å in B, respectively. The values of the valence angles on the S atom change from 103.13(15)° for O8A—S6A—N5A in A to 122.1(2)° for O7B—S6B—O8B in B, which shows clear deviations from the ideal tetrahedral configuration on the S atom with the values of the valence angles of 109°. These deviations are related to the mutual repulsion of lone pairs on the oxygen atoms, which causes them to be located as far away from each other as possible.

The sulfonylaminothiourea chain adopts trans-cis-gauche-gauche conformation in both molecules with similar values of the torsion angles C21–N1–C2–N4, N1–C2–N4–N5, C2–N4–N5–S6 and N4–N5–S6–C31 of 178.2(3), −11.8(4), 123.1(3) and 55.7(2)° for A and −173.2(3), −7.5(5), 108.2(3) and 56.4(2)° for B, respectively. The thione C2–S3 group has cis and trans conformation with respect to N1–C2 and N4–C2 bonds, respectively, as shown by the torsion angles for C21–N1–C2–S3 of −4.6(5)° and N5–N4–C2–S3 of 175.8(2)° in A and 6.7(5) and 172.6(2)°, respectively, in B. The torsion angles for N4–N5–S6–O7 and N4–N5–S6–O8 of −60.8(2) and 170.3(2)° in A and 171.6(2) and −59.1(3)° in B, respectively, show that the S=O groups of the sulfonyl part of the sulfonylaminothiourea chain adopt trans and gauche conformation with respect to the N5–S6 bond in both molecules. Molecules A and B differ in the position of the benzene and cyclohexane rings relative to the sulfonylaminothiourea spacer, as shown by the torsion angles for N5–S6–C31–C32 and C2–N1–C21–C22 of −113.9(3) and −113.7(4)° in A and 82.6(3) and 133.9(4)° in B, respectively. In both molecules, the hexane rings adopt an almost ideal chair conformation with puckering parameters [34] of Q = 0.563(4) Å and θ = 178.8(4)° for A and Q = 0.559(4) Å and θ = 0.0(4)° for B. The conformations of the A and B molecules are influenced by the intramolecular hydrogen bond N1–H1…N5 (Figure 1, Table 1). Moreover, the molecules form a molecular dimer using the pair of N4–H4…S3 intermolecular hydrogen bonds (Table 1).

Identification of intermolecular interactions in the crystalline state is crucial from the point of view of potential interactions of a biologically active molecule in the active site of a potential molecular target. In the crystal structure of WZ1 the packing of the molecules is influenced by the intermolecular hydrogen bonds N5A–H5A…S3A and N5B–H5B…S3B (Table 1), which link the molecular dimers formed by molecules A and B related by the 2_1_ symmetry axis into molecular chains parallel to the Y direction. 

### 2.3. In Silico Prediction of Cytotoxicity for Cancer

To investigate the anticancer benefits of new synthesized compounds, Cell Line Cytotoxicity Predictor (CLC-Pred) 2.0 was used [35]. CLC-Pred 2.0 is a website used to predict in silico the cytotoxic effects of chemical compounds based on their structural formula. CLC-Pred 2.0 makes it possible to predict the toxicity towards cell lines of the tested compound in order to assess the appropriateness of including the substance in an in vitro test. This allows it to predict the cytotoxicity of tested compounds. Table 2 shows the results of the in silico predicted (Pa > 0.5) anticancer activity for the compounds WZ1–WZ4.

The analysis showed the highest potential activity of the tested compound against the HepG2 and DU-145 cell lines, representing hepatoblastoma and prostate cancer, respectively. In addition, this service was used to predict the potential mechanism of molecular activity for the given compounds (Table 3, for Pa > 0.6). For each compound, inhibition of Aldehyde dehydrogenase 1A1 is the most likely predicted mechanism of biological activity. 

### 2.4. ADMET Analysis

Pharmacokinetic parameters (ADMET) play an important role in the process of designing and synthesizing new drugs. High efficacy against the therapeutic target with favorable ADME parameters characterize a good drug candidate [36,37]. The BOILED-Egg model was used to predict gastrointestinal absorption [38], and the toxicities were predicted using the ProTOX II service [39] for WZ1–WZ4.

**Figure 3:**
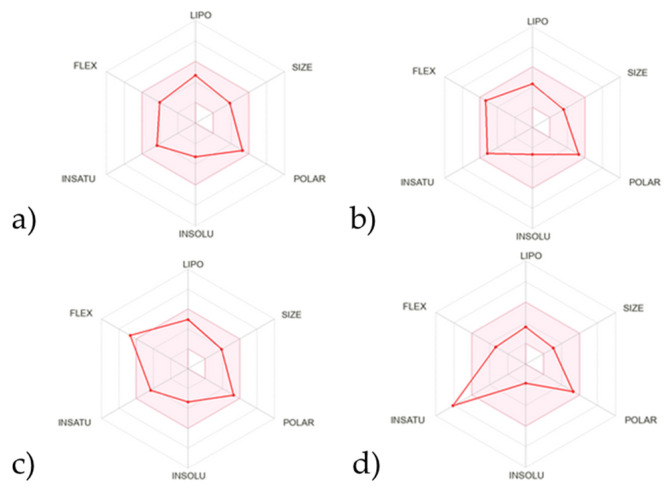
Bioavailability radar for (**a**) WZ1, (**b**) WZ2, (**c**) WZ3 and (**d**) WZ4. (LIPO—lipophilicity is within the range −0.7 < XlogP3 < +5.0; SIZE—molecular weight is 150 g/mol < MW < 500 g/mol; POLAR—polarity is 20 Å < TPSA < 130 Å; INSOLU—insolubility is 0 < logS < 6; INSATU—insaturation is 0.25 < Fraction Csp3 < 1; FLEX—Flexibility is 0 < Num. rotatable bonds < 9). Table 4. The putative prediction drug–drug interactions via cytochrome P450 (CYP) for WZ1–WZ4.

**Figure 4:**
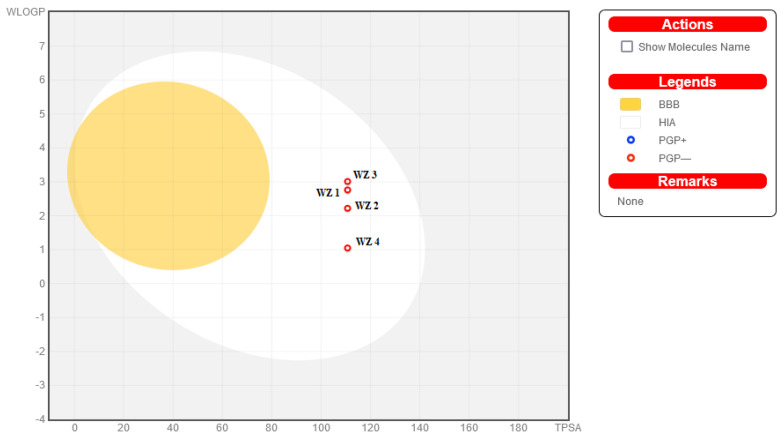
BOILED-Egg diagram for WZ1–WZ4.

All obtained compounds (WZ1–WZ4) are characterized by high absorption in the gastrointestinal tract, which makes them possibly attractive drug candidates. Their high potential against multidrug-resistant cancer cells also confirms that they do not act as a P-gp substrate [40]. Figure 3 shows the bioavailability radars for WZ1–WZ4. The WZ1 and WZ2 compounds have appropriate physicochemical properties for oral bioavailability (they are included in the colored zone on the radar). The compound WZ3 has too many rotatable bonds and WZ4 has too low a Csp 3 fraction coefficient corresponding to unsaturation, which means that they slightly exceed the required physicochemical properties for oral bioavailability. Moreover, they have a good bioavailability index (0.55) [41] and meet all drug-likeness rules such as those of Lipiński [42], Ghose [43], Egan [44], Veber [45] and Muegge [46]. Figure 4 shows the BOILED-Egg diagram; all of the tested compounds are able to be absorbed gastrointestinally. Prediction of putative drug–drug interaction by cytochrome P450 (CYP) inhibition is presented in Table 4. Thus, the presented ADME parameters indicate the promising nature of the designed compounds.

Servis ProTox II indicated the fourth toxicity class for each compound with a different LD_50_, WZ1 and WZ3 (predicted LD_50_: 2000 mg/kg for both), WZ2 (predicted LD_50_: 1000 mg/kg) and WZ4 (predicted LD_50_: 750 mg/kg). Moreover, tested compounds are not immunotoxic, which makes them quite safe and promising for further research.

### 2.5. Molecular Docking Results

**Figure 5:**
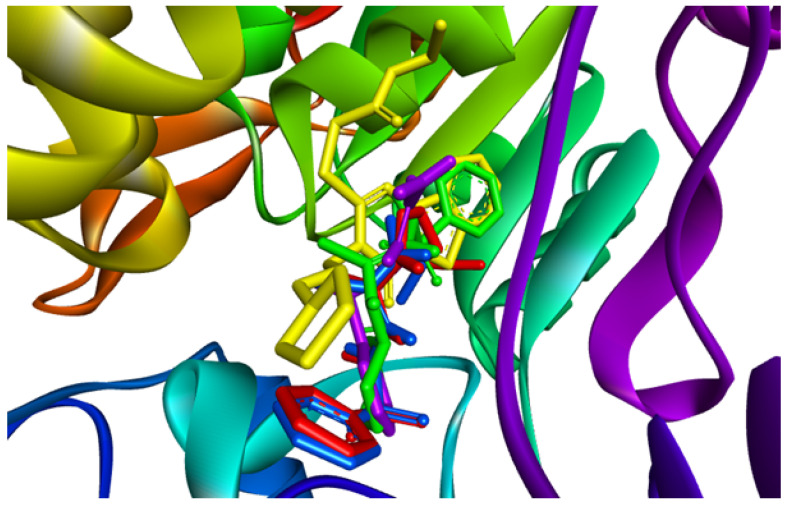
Superimposed image of WZ1 (green), WZ2 (blue), WZ3 (red), WZ4 (purple) and native ligand (yellow) in the binding cavity.

Docking simulation was performed for the tested WZ1–WZ4 compounds to obtain a comprehensive understanding of the likely modes of action and quantification of anticancer activity. Docking scores are listed in Table 5. A superimposed diagram of WZ1–WZ4 and native ligand in the binding cavity is presented in Figure 5. Binding energy analysis revealed that WZ1 was supposed to be the most active against ALDH1A1. Docking scores confirmed the possible anticancer activity of the synthesized ligands.

**Figure 6:**
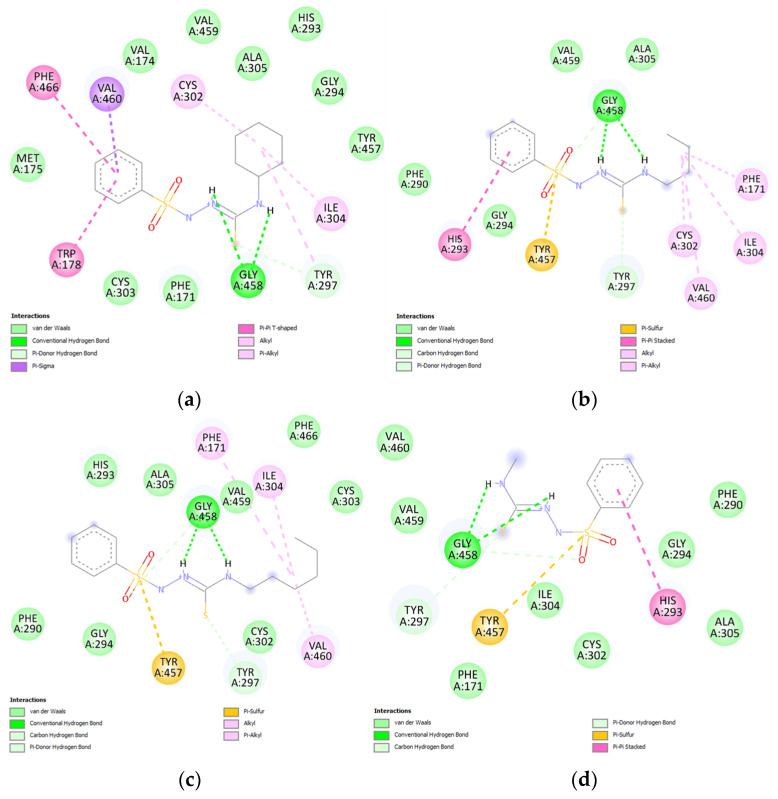
A 2D scheme of the ALDH1A1 complexes with (**a**) WZ1, (**b**) WZ2, (**c**) WZ3 and (**d**) WZ4.

All of the tested compounds created conventional hydrogen bonds with GLY458 by NH groups of the thiosemicarbazide scaffold (Figure 6). This is the main interaction stabilizing the protein–ligand complex. Moreover, for the aromatic groups of WZ1, WZ2 and WZ4 different types of Pi interactions were observed. Analysis of the docked poses indicated a different location of the WZ4 compound. Its phenylsulphonic group is located in a place where the isothiocyanate chains of compounds WZ1–WZ3 are located. The variation of the pose may be related to the size of the compound, WZ4 is the smallest compound from the tested series. The analysis of the binding energy values indicated that replacing the cyclohexyl ring originating from isothiocyanate with an alkyl one and extending the chain result in a weaker interaction with the protein binding cavity.

### 2.6. The Antimicrobial Activity Assessment

**Figure 7:**
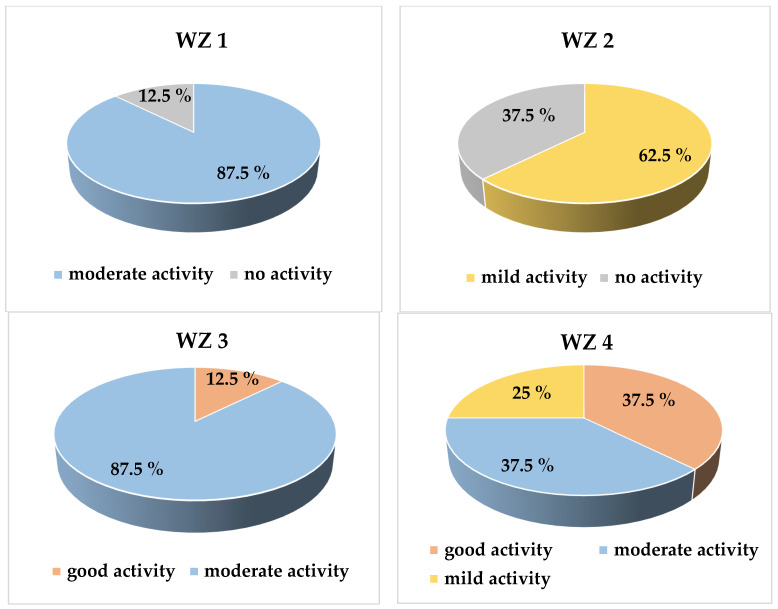
The bioactivity data expressed as antimicrobial potency of the compounds WZ1–WZ4 towards reference Gram-positive bacteria (*Staphylococcus* spp., *Micrococcus* spp., *Enterococcus* spp. and *Bacillus* spp.) used in the study (good activity—MIC in the range 26–125 µg/mL; moderate activity—MIC in the range 126–500 µg/mL; mild activity—MIC in the range 501–1000 µg/mL; no activity—MIC > 1000 µg/mL).

The data presented in Table 6 and Figure 7 indicated that compounds WZ1–WZ4 possessed potential antimicrobial activity. Their lowest concentrations inhibiting the growth of the reference microorganisms (MIC—minimum inhibitory concentration) ranged from 62.5 to ≥2000 µg/mL. In turn, the lowest kill concentrations (MBC or MFC—minimum bactericidal or fungicidal concentration) were within the range from 125 to ≥2000 µg/mL. They indicated higher effectiveness against Gram-positive bacteria than Gram-negative and yeasts. Among Gram-positive microorganisms, reference strains of *Staphylococcus epidermidis, Bacillus subtilis* and *Bacillus cereus* were most sensitive to the studied compounds, especially to WZ4 (MIC = 62.5–125 µg/mL). This confirms the good activity of WZ4 towards them. This new compound showed also moderate activity towards *Staphylococcus aureus* ATCC 43300, *Staphylococcus aureus* ATCC 25923 and *Micrococcus luteus* (MIC = 250–500 µg/mL). In turn, *S. aureus* ATCC 29213 and *Enterococcus faecalis* were slightly less sensitive and WZ4 indicated a mild effect towards them (MIC = 1000 µg/mL). The compound WZ3 showed also a good effect towards *B. subtilis* (MIC—125 µg/mL) and a moderate effect to the remaining Gram-positive microorganisms with MIC = 250–500 µg/mL. Additionally, the MBC/MIC ratio of compounds WZ3 and WZ4 towards most Gram-positive bacteria was in the range 1–4, indicating the bactericidal effect of them. Moreover, a good effect was found for the compound WZ1 against these bacteria (MIC = 250–500 µg/mL), except for *E. faecalis* (MIC = 2000 µg/mL). WZ2 showed mild activity towards most Gram-positive bacteria (MIC = 1000 µg/mL) and no bioactivity against three strains of cocci: *S. aureus* ATCC 43300, *S. aureus* ATCC 29213 and *E. faecalis.* Gram-negative rods from *Enterobacterales* and non-fermenting bacteria (*Pseudomonas aeruginosa*) were insensitive to the studied compounds, except for substance **WZ3** (MIC = 500–1000 µg/mL). The antibacterial effect of **WZ3** was moderate against *Bordetella bronchiseptica* and *Escherichia coli* with MIC = 500 µg/mL and mild towards the remaining Gram-negative bacteria (MIC = 1000 µg/mL).

In the case of fungi, belonging to reference *Candida* spp., different activity was indicated. These compounds showed an antifungal effect with MIC = 125–2000 µg/mL (WZ3 and WZ4) or no anticandidal bioactivity (WZ1 and WZ2). Among them, the most active compound was WZ4 with a good effect against *C. parapsilosis* and *C. glabrata* (MIC = 125 µg/mL). In turn, the activity of the both compounds WZ3 and WZ4 towards *C. albicans* and *C. krusei* (MIC = 250–500 µg/mL) was moderate. MFC values of both compounds towards yeasts were similar (same as MIC or 2–8-fold higher) and their range was 500–2000 µg/mL. These compounds indicated a fungicidal effect against reference yeasts (with MFC/MIC = 1–4), except WZ4 towards *C. parapsilosis* (fungistatic effect with MFC/MIC = 8).

In conclusion, the results from Figure 7 show good bioactivity of compounds WZ3 and WZ4 towards 12.5% and 37.5% of the reference strains of Gram-positive bacteria, respectively. Moreover, the newly synthesized compounds (except WZ2) indicated a mainly moderate activity against 87.5% (compounds WZ1 and WZ3) and 37.5% (compound WZ4) of these microorganisms. The mild effect was also shown by WZ2 (62.5%) and WZ4 (25%). However, some Gram-positive bacteria were not susceptible to compounds WZ1 (12.5%) and WZ2 (37.5%). 

### 2.7. The Hemolytic Activity Assay

**Figure 8:**
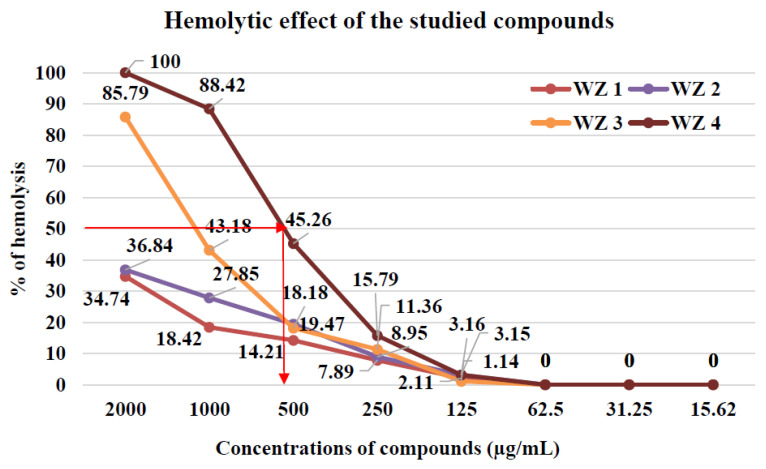
Hemolytic effect (% of hemolysis) of the compounds WZ1–WZ4.

Our results revealed that studied compounds exhibit negligible toxicity, as compared to the positive control Triton X-100 (100% erythrocyte lysis). As presented in Figure 8, hemolytic activity of each compound was related to their concentration. The compounds with a mild antibacterial effect (MIC = 1000 µg/mL) showed some hemolytic activity in the range 18.42–88.42%. On the other hand, with their concentration ≤ 500 µg/mL, the percentage of lysed red blood cells ranged from 0 to 45.26, which was dependent on the compound. The activity of compounds WZ1, WZ3 and WZ4 was moderate or good against almost all Gram-positive bacteria (MIC = 62.5–500 µg/mL) and data obtained using ELA confirmed that this antimicrobial effect was observed at their < 50% hemolytic activity. The concentrations ≤ 125 µg/mL of all compounds did not affect the stability of the erythrocyte membrane (<5% erythrocyte lysis) [47].

### 2.8. In Vitro Cell Assay

**Figure 9:**
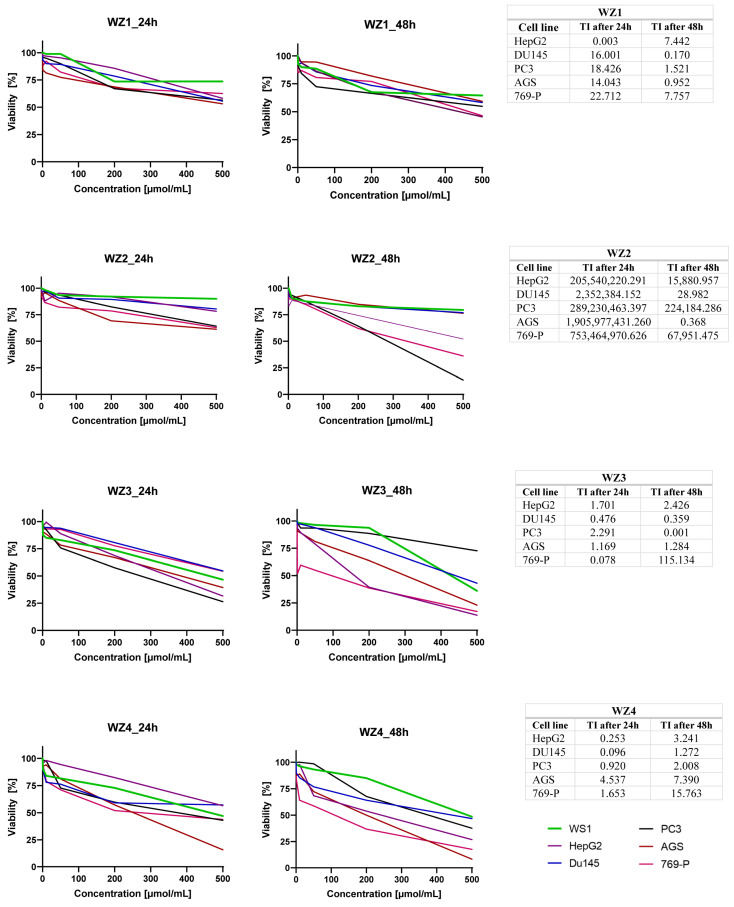
Effect of 24 h and 48 h exposure of WZ1–WZ4 on the percentage of cell viability of WS1 (green line), 769-P (pink line), PC3 (black line), HepG2 (purple line), AGS (red line) and DU145 (blue line) by MTT assay. TI—Therapeutic index (ratio of the IC_50_ of physiological cells to the IC_50_ of tumor cells).

To measure the metabolic activity of cells, the MTT assay was used as an indicator of cell viability and cytotoxicity. WZ1–WZ4 were shown to reduce the viability of both prostate cancer lines (Du145 and PC3), as well as hepatocellular carcinoma lines (HepG2), gastric cancer (AGS) and renal adenocarcinoma (769-P). Data are presented in Figure 9.

For WZ1, after both 24 h and 48 h, a decrease in viability of the physiological line at the level of statistical significance was observed at a concentration of 200 µM (*p* < 0.0001). At the same concentration, a decrease in viability of ~15% (*p* = 0.0042) of HepG2 was observed after 24 h of incubation, while prolonging the incubation time decreased HepG2 viability ~7% already at a concentration of 10 µM (*p* = 0.0190). After 24 h of incubation with WZ1 for prostate cancer, both Du145 and PC3 showed a significant reduction in viability already at concentrations of 1 µM and 50 µM, respectively (*p* < 0.05; *p* = 0.0006) (~7% and ~11%, respectively). In contrast, the longer 48 h incubation shifted the significant reduction in viability towards higher concentrations for Du145 (200 µM; *p* < 0.0001) and towards lower concentrations for PC3 (10 µM; *p* < 0.0004). In the case of the 769-P line, a significant decrease in viability was already observed at a WZ1 concentration of 1 µM during both 24 h and 48 h incubations, i.e.: ~11% and ~15% (*p* < 0.0001). For the AGS line, a statistically significant cytotoxic effect was observed after 24 h at ~16% (*p* < 0.0001) for the concentration of 1 µM and after 48 incubation at ~6% for the concentration of 50 µM *(p* = 0.0302).

For WZ2, after 24 h of incubation, no reduction in viability was observed against WS1 over the concentration range tested (1–500 µM). In contrast, after 48 h, a reduction in their viability of ~17% (*p* = 0.0238) was observed at a concentration of 200 µM, and of ~23% at a concentration of 500 µM (*p* = 0.0009). After 24 h incubation with the same compound at a concentration of 500 µM, a reduction in HepG2 viability by ~22% (*p* < 0.0001) was observed. In contrast, after just 48 h of incubation, the viability of HepG2 cells decreased to ~82% at a concentration of 1 µM (*p* < 0.0001). For the Du145 line, after 24 h their viability dropped to ~80% (*p* = 0.0007) at a concentration of 50 µM and after 48 h to ~92% at a concentration of 1 µM (*p* = 0.0051). While for the PC3 line, a reduction in viability to ~82% was observed at a concentration of 200 µM (*p* = 0.0001), and incubation for 48 h shifted this value towards lower concentrations (50 µM; ~88%; *p* = 0.0097). In contrast, for line 769-P, a cytotoxic effect was observed at a concentration of 1 µM at ~8% (*p* = 0.0450) after 24 h and ~10% *(p* < 0.0001) after 48 h. The same concentration decreased the viability of the AGS line by ~8% (*p* = 0.0004) after 24 h and by ~6% (*p* = 0.0010) after 48 h of incubation with WZ2.

The compound WZ3 statistically significantly decreased the viability of the physiological line after 24 h incubation across the range of concentrations tested (1–500 µM) (*p* < 0.0001). In contrast, increasing the incubation time contributed no significant change in viability in the concentration range 1–200 µM (*p* > 0.05). For the HepG2 line, a statistically significant cytotoxic effect of ~31% was observed after 24 h incubation (*p* < 0.0001) at a concentration of 200 µM. At the same concentration, a cytotoxic effect was also observed for the Du145 line, where viability decreased by ~19% (*p* < 0.0001). A 48 h incubation with WZ3 at a concentration of 1 µM significantly decreased HepG2 viability by ~7% (*p* = 0.0012), and at a concentration of 50 µM there was a significant decrease in Du145 viability by ~6% (*p* = 0.0384). The same concentration of WZ3 after 24 h of incubation reduced the viability of the second prostate cancer line PC3 to ~76% (*p* < 0.0001). A significant reduction in viability of ~11% after 48 h incubation for the PC3 line was observed at a concentration of 200 µM (*p* = 0.0184), and after 24 h incubation for the 769-P line the same concentration reduced viability by ~22% (*p* < 0.0001). After 48 h, a decrease in viability of ~48% was observed for the 769-P line at a concentration of 1 µM after 48 h of incubation (*p* < 0.0001). In contrast, for the AGS cell line, the lowest concentration (1 µM) of WZ3 used after both 24 and 48 h incubation significantly reduced viability by ~9% (*p* = 0.0002; *p* < 0.0001).

For compound WZ4, a significant decrease in viability was observed across the range of concentrations tested (1–500 µM) (*p* = 0.0189; *p* < 0.0001) after 24 h incubation for the fibroblast cell line (WS1). While, increasing the incubation time decreased the viability of WS1 statistically significantly at a concentration of 200 µM (*p* = 0.0027) by ~15%. The same concentration reduced HepG2 viability by ~18% after 24 h (*p* = 0.0002), while a concentration of 50 µM after 48 h incubation reduced HepG2 viability to ~69% (*p* < 0.0001). Relative to the Du145 line, WZ4 significantly reduced viability over the entire range of concentrations tested (1–500 µM), both after 24 h and 48 h incubation (*p* = 0.0012; *p* < 0.0001). In contrast, for the second prostate cancer line PC3, a significant cytotoxic effect after 24 h incubation was observed at a concentration of 50 µM (~27%), and after 48 h at a concentration of 200 µM (~32%) (*p* < 0.0001). For the 769-P line, after 48 h of incubation with WZ4, there was also a significant decrease in viability of ~16–82% across the range of concentrations tested (1–500 µM), and after 24 h in the concentration range of 10–500 µM (~21–56%) (*p* < 0.0001). For the gastric cancer AGS cell line, significant decreases in viability were observed by ~7–84% over the entire range of concentrations tested (1–500 µM) after 24 h and by ~12–92% after 48 h (*p* < 0.0001).

### 2.9. Determination of Concentration Aldehyde Dehydrogenase 1 Family, Member A1 (ALDH1A1)

**Figure 10:**
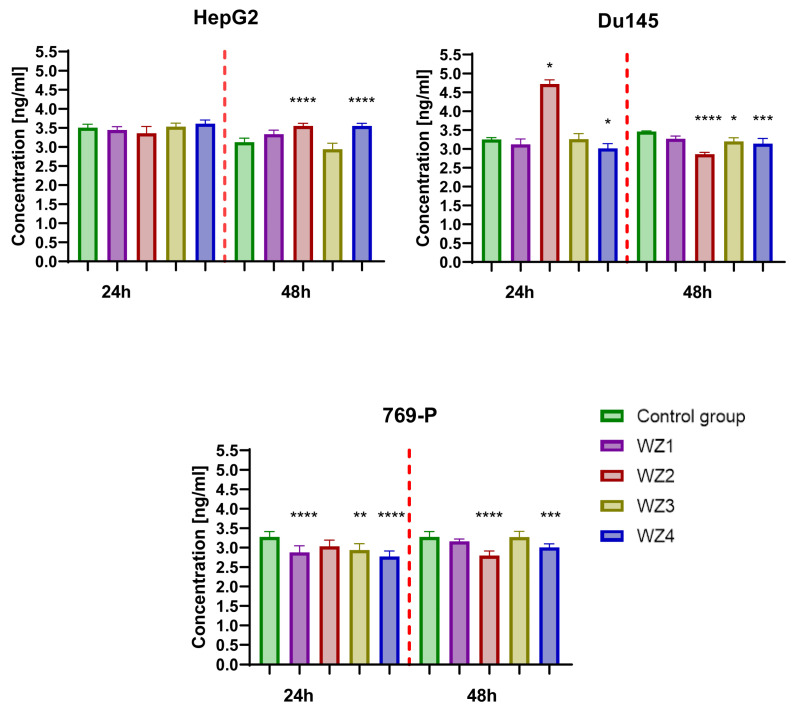
Effects of incubation with WZ1-WZ4 compounds on ALDH1A1 levels in selected cell lines after 24 h and 48 h. *p* < 0.05 *; *p* < 0.01 **; *p* < 0.001 ***; *p* < 0.0001 ****. The significance level was taken as *p* = 0.05. The graph present mean with the standard deviation (SD).

The effect of compounds WZ1–WZ4 on ALDHA1 levels was investigated (Figure 10). The Du145, HepG2 and 769-P cell lines were treated with the calculated IC_25_ values for each compound. 

For the HepG2 cell line, no significant changes were observed, as compared to the control after 24 h of incubation. Extending the incubation time to 48 h resulted in an increase in ALDH1A1 concentration for the WZ2- and WZ4-treated groups compared to controls (*p* < 0.0001). Also, 24 h incubation of WZ2 with the prostate cancer line (Du145) increased ALDH1A1 levels in cells compared to the control group (*p* < 0.05). In contrast, prolonging this time resulted in a decrease in the concentration of the enzyme compared to the control group (*p* < 0.0001). For 24 h and 48 h incubation of WZ4 with Du145, a decrease in ALDH1A1 concentration was observed compared to the control (*p* < 0.05; *p* < 0.001). For compound WZ3, a decrease in the enzyme concentration in cells was observed only after 48 h of incubation (*p* < 0.05). A decrease in ALDH1A1 levels was observed, as compared to the control, after 24 h of incubation at the level of statistical significance for the 769-P cell line for WZ1, and an increase was observed for WZ3. On the other hand, for WZ4, a decrease in activity was observed after both 24 h and 48 h of incubation, as compared to the control. However, in the case of WZ2, no significant changes were observed within the 24 h incubation of the compound with the 769-P cell line, but prolonged incubation resulted in a significant decrease in ALDH1A1 activity.

## 3. Discussion

As a result of numerous studies, a significant variety of bioactive heterocyclic molecules have been developed. Among them, great interest due to their biological activity was devoted to thiosemicarbazides, compounds containing sulfur and nitrogen [48,49,50]. The literature data indicate a strong structure–activity relationship, and the type of substituent and location of the substituent are crucial for the activity of the compound. It was shown that thiosemicarbazide derivatives with a 4-nitrophenyl group exhibited an antibacterial effect towards staphylococci *S. aureus* and *S. epidermidis*; streptococci *S. mutans* and *S. sanguinis*; moderate cytotoxicity and good therapeutic safety in vitro [27]. Other authors evaluated the activity of (-)-camphene-based thiosemicarbazide (TSC) and 4-hydroxy-thiosemicarbazone (4-OH-TSZ) against some Gram-positive bacteria: *S. aureus* and *Enterococcus* spp. [51]. Their results showed potential bactericidal activity of the both compounds towards these bacteria, including multidrug-resistant strains (with MIC in the range from 1.9 to 31.2 μg/mL). Moreover, other thiosemicarbazide derivatives (thiophene-1,2,4-triazole-5(3)-ones) also demonstrated antimicrobial effects against *S. aureus*, *Mycobacterium smegmatis* and *B. cereus* [52]. Similarly, our research results revealed the activity of new derivatives, especially WZ4, against *B. cereus*. It is worth noting that the tested compounds have a sulphonic group in their grouping. This is one of the reasons that allows us to make conclusions about the potential antibacterial activity of the tested compounds. Furthermore, one study showed that the antimicrobial activity of aliphatic sulphonamides decreased with increasing carbon chain length [53]. This compound has the shortest aliphatic chain of all the compounds tested, i.e., the methyl group. *B. cereus* is mainly found in contaminated food. It is usually the cause of diarrhoal poisoning, but also opportunistic infections [54]. The important consideration is that it is a spore-producing bacterium. The spores can survive various unfavorable conditions, e.g., high temperatures during pasteurization or spray-drying of milk. It is therefore important to eliminate them from the food supply. In the case of compound WZ4, it should be investigated in the future whether it inhibits both vegetative cells and spores of this microorganism. The antifungal activity of thiosemicarbazides has been investigated in a number of studies [55,56,57]. The linking active substructure method was used to synthesize a series of novel benzaldehyde thiosemicarbazide derivatives containing heterocyclic piperidine [58]. These compounds exhibited antifungal activity, especially against *Valsa mali, Rhizoctonia solani*, *Pythium aphanidermatum* and *Gaeu-mannomyces graminsis*. It is worth noting that in another study, two isoquinoline derivatives of thiosemicarbazide with an ortho-methyl group in the phenyl ring also indicated the potential anticandidal effect [59]. These compounds were effective against *C. albicans* and *C. parapsilosis*. Therefore, it seems that the number of methyl groups and their nature may be crucial for the antifungal activity.

Overall, the Gram-positive bacteria were more susceptible to the newly synthesized compounds WZ1–WZ4 than the Gram-negative and yeasts belonging to *Candida.* The degree of sensitivity of these microorganisms to the tested agents may be related to the different structures of their cell wall. The cell wall of Gram-positive bacteria consists of many layers of peptidoglycans composed of N-acetyl-muramic acid, polysaccharides and N-acetyl-glucosamine connected by cross-bridges and peptide chains [60]. Perhaps, the active compounds break important bonds in their cell wall more easily. In turn, the structure of the cell wall of Gram-negative bacteria is much more extensive, which make them more resistant to the studied compounds.

In the present studies, the toxicity of compounds WZ1–WZ4 towards erythrocytes was assessed using the hemolytic test in vitro. The blood red cells were used to analyze the effect of these substances on their cell membrane. It should be noted that the membranes of erythrocytes and other body cells are similar. Hemolysis is the destruction of red blood cells by lysis of the membrane lipid bilayer. Its results can change depending on the type and concentration of the tested compounds [61]. These data may reveal some information about the mechanism of cytotoxicity of the tested compounds. Moreover, the erythrocyte model presents a direct indication of toxicity of the injectable formulations. The results of the present study showed that the compounds WZ1 and WZ2 indicate a lower hemolytic effect as compared to WZ3 and WZ4. On the other hand, literature data related to the evaluation of thiosemicarbazide toxicity on the erythrocyte membrane are limited. It is worth noting that also in relation to studies on physiological cells (WS1), similar results were obtained. Compounds WZ3 and WZ4 showed lower TI compared to WZ1 and WZ2.

The search for new anticancer drugs is a multi-step process. One of them is in vitro research using cell lines. Its undoubted advantage is simplicity, cost-effectiveness and compliance with the 3Rs principle [62,63]. These studies are screening platforms for the initial evaluation of new substances. 

The following cancer cell lines were selected for the study: HepG2, Du145, PC3, AGS and 769-P. The physiological human fibroblast line WS1 was selected to assess the selectivity of action. Based on the MTT test, the IC_50_ of the test compounds was determined, and in the next step, the TI was calculated. The calculated values show how many times the determined IC_50_ on the tumor line is lower than on the physiological line. Thus, if the TI is higher, the compound is characterized by a higher selectivity and cytotoxic activity in cancer cells. Analyzing the calculated TI, the compound WZ2 had the highest selectivity. In its case, no significant decrease in viability was observed for the physiological line at the tested concentrations after 24 h of incubation. However, after increasing the incubation time of the compound with WS1, a significant decrease in viability was observed at a concentration of 200 µM. It is notable that increasing the incubation time of the compound with the tumor lines also shifted the observed decrease towards lower concentrations. For the HepG2 cell line, after 24 h a significant decrease in viability was observed at the highest concentration tested (500 µM), while after 48 h it was already observed at the lowest concentration tested, 1 uM. The concentration of 1 µM decreased the viability significantly for other lines: DU145 (48h), 769-P and AGS.

The IC_50_ values for almost all lines tested were <1000 times lower than those IC_50_ values calculated for line WS1. Only at 48 h for the AGS line was the TI relatively low. Therefore, WZ2 appears to be the best candidate with cytotoxic properties among the compounds tested. The high selectivity against almost all tested tumor lines provides grounds for extending the study to other tumor lines and for a more in-depth investigation of its mechanism of action. Its activity may be due to the ‘optimal’ length of the carbon chain, which favors its solubility, while being long enough to penetrate lipid membranes and exert its effects in cells (the longer the aliphatic chain, the more it resembles cell membrane lipids). In contrast, compounds WZ3 and WZ4 had the lowest selectivity. Comparing the TI values, these coefficients appear quite low as compared to WZ1 and WZ2. In the case of compound WZ1, in almost every test line, extending the incubation period to 48 h reduced its selectivity of action (except HepG2). The compound WZ1, only at a concentration of 200 µM, significantly statistically decreased the viability of the physiological line WS1. However, the same concentration also caused a significant decrease in viability for the HepG2 (24 h) and DU145 (48 h) lines. At lower concentrations, it significantly decreased viability after 24 h for both prostate lines, after both 24 h and 48 h for the gastric cancer line (AGS) and 769-P, and after 48 h for the HepG2 line.

Interestingly, and in need of further study, prolonging the incubation time of WZ1 with cells worsened its selectivity of action. It is the only compound having an aliphatic chain that has undergone cyclization. This may be an effect of chain cyclization, as such an effect was not observed for compound WZ3. Almost all compounds had cytotoxic effects with selectivity against renal adenocarcinoma lines, except WZ3 after 24 h. However, WZ3 showed selectivity against this line after 48 h with TI = 115.134. It showed negligible/no TI < 1 selectivity against the DU145, PC3 line (after 48 h). WZ3 significantly reduced the viability of the physiological line at the lowest tested concentration of 1 µM after 24 h of incubation by ~12%; however, extending this time only significantly reduced the viability of these cells at the highest tested concentration of 500 µM by ~64%. The lowest tested concentration also significantly decreased the viability of the tumor lines, i.e., HepG2 by ~7% (48 h); 769-P by ~22% (24 h) and ~ 48% (24 h; 48 h); and AGS by ~9% (24 h, 48 h). The relatively high toxicity may also be because WZ3 has the longest aliphatic chain, which may make it easier to penetrate cell lipid membranes. Moreover, in future research directions, it would be worthwhile to look into the cyclization effect of aliphatic chains.

Similarly, compound WZ4 did not show satisfactory TI < 1 selectivity against both prostate cancer lines (24 h) and HepG2 (24 h). It is noteworthy that this selectivity improved after prolonged incubation of these cells with the compound (10 < TI > 1). Similar to WZ3, compound WZ4 reduced the viability of human fibroblast lines at all concentrations tested (~10–52%). After 48 h incubation with this compound, a significant decrease in viability of ~15% was only observed at a concentration of 200 µM. With this compound, several tumor lines showed a significant reduction in viability across the range of concentrations tested. The lowest concentration of 1 µM significantly reduced viability: ~10% (DU145 at 24 h; 48 h); ~16% (769-P at 48 h); ~7% (AGS at 24 h) and ~12% (AGS at 48 h). Only for the hepatocellular carcinoma lines were no significant decreases in viability observed at both incubation times (~4% at 24 h; ~3% at 48 h). WZ4 appears to have the least selectivity of action and seems to have the least anticancer potential. The low selectivity may be due to the short carbon chain, which makes the compound more soluble, more rapidly metabolized, and therefore more toxic. The implication is that only compounds with the correct chain length and structure will pass through the cell membrane. Thiosemicarbazides with a sulfone group have never been tested for their anti-inflammatory effects, but they have been shown to have significant anti-tuberculosis effects [31]. As research has shown, these compounds have broad biological activity, but the molecule itself is also toxic [64,65]. A key feature is that the introduction of a sulfonic group into the thiosemicarbazide reduces the toxicity of the various combinations, and can even, in some cases, eliminate the toxic effect of the molecule. The reason for this is probably increasing in their solubility, which makes them more easily metabolized and excreted from the organism.

Compounds WZ3 and WZ4 have hexyl and methyl substituents in their molecule, respectively, compound WZ1 has a cyclohexyl group in its molecule, and WZ2 has a butyl group. These compounds also differ in the length of the carbon chain in the molecule. The WZ4 molecule possesses a higher toxicity, which may be related to the shorter aliphatic chain in the molecule (-CH_3_) than the other tested compounds. In the case of WZ1, the combination of cyclohexyl into the molecule appears to have reduced toxicity to human fibroblasts, as compared to the structure and mechanism of action of WZ3.

ALDH1A1 is thought to play an important role in tumorigenesis and progression [66,67,68]. The cell lines proposed by CLC-Pred 2.0 (HepG2 and DU145), but also the 769-P line, against which the compounds showed the highest activity, were selected for marking ALDH1A1 levels. High ALDH activity is one of the prognostic markers for prostate cancer. Cells with increased ALDH1A1 expression also show high resistance to radiotherapy [69]. In prostate cancer, the combination of ALDH1A1 inhibitors with other conventional tumor therapies is believed to be a promising strategy in the development of anticancer therapies [70]. Our study showed that after 48 h of incubation with compounds WZ2, WZ3 and WZ4, ALDH1A1 was significantly reduced compared to the control. In the case of the prostate cancer cell line DU145, a decrease in ALDH1A1 levels was observed in the cells after 48 h of their incubation with WZ2–WZ4. A similar effect was also observed for the renal adenocarcinoma line 769-P. A 24 h incubation with WZ1, WZ3 and WZ4 decreased the levels of the enzyme. Whereas, prolonging this time resulted in a significant decrease in ALDH1A1 levels only for WZ2 and WZ4. This also corresponds with the fact that these compounds also had a cytotoxic effect on DU145 cells. Considering the TI values calculated for the compounds, as well as the ALDH1A1 values, the most promising compound is with anti-tumor properties against the DU145 line is WZ2. In the case of renal adenocarcinoma (769-P), where compounds showed the greatest activity and selectivity, a significant reduction in ALDH1A1 was observed for almost all tested compounds. As with the DU145 line, the most promising compound is WZ2, followed by WZ1 and WZ4 in terms of anti-tumor effect towards 769-P. For this tumor, it has also been shown that over-repression of this enzyme is associated with shorter recurrence-free survival and overall survival, as well as greater invasion and proliferation of neoplastic cells [71,72]. Therefore, it is considered that ALDH1A1 may be an important target for anti-tumor therapies for renal adenocarcinoma. 

Importantly, ALDH1A1 may also act as a tumor suppressor. In our study, we found increased levels of ALDH1A1 for hepatocellular carcinoma cell lines (HepG2) after treatment with WZ2 and WZ4, after 48 h of incubation. The shorter incubation time did not induce any significant changes in ALDH1A1 levels in the cells tested. The WZ2 compound also appears to be of interest in the context of its anti-tumor effect towards hepatocellular carcinoma (HepG2). It combines a high TI while affecting one of the mechanisms involved in carcinogenesis. Slightly worse is the compound WZ4, which has a lower TI and therefore lower selectivity, but affects significantly increased ALDH1A1 levels in cells.

It is also important to note that ALDH1A1 is highly expressed in liver tissue [73]. One study showed that high levels of ALDH1A1 were associated with low serum α-fetoprotein levels, small tumor diameter and very little lymphatic vessel invasion in hepatocellular carcinoma [74]. Another study found that high levels of ALDH1A1 were associated with longer survival in hepatocellular carcinoma [75]. It is worth emphasizing here that our studies showed that both compounds (WZ2 and WZ4) reduced the viability of HepG2 cells in the MTT test. Therefore, the results of our research confirm and largely complement existing work on new derivatives and indicate the promising cytotoxic potential of these compounds.

## 4. Materials and Methods

### 4.1. Chemistry

All reagents used for the synthesis were purchased from AlfaAesar (Haverhill, MA, USA), Sigma-Aldrich (Saint Louis, MO, USA) and POCH (Haverhill, MA, USA) and used without purification.

#### 4.1.1. General Synthesis Procedure for N-Substituted 2-(Benzenosulfonyl)-1-Carbotioamide Derivatives WZ1–WZ4

A mixture of 0.0l moles (1.72 g) of benzenesulfonohydrazide WZ and 0.01 moles of appropriate isothiocyanate in 10 mL of anhydrous ethanol was kept at boiling for 1–2 h. The formed compound was filtered off, washed and crystallized from ethanol. 

The obtained compounds were identified by analyzing ^1^H and ^13^C NMR, IR and MS spectra. The melting point on the Fisher-Johns block was also determined. The Bruker Avance 600 spectrometer (Bruker BioSpin GmbH, Rheinstetten, Germany) was used to record ^1^H and ^l3^C NMR spectra in DMSO-d_6_. Chemical shifts are given on a scale of δ ppm (parts per million) relative to an inertial standard (TMS) or residual solvent peak. A Thermo Scientific Nicolet 6700 FTIR spectrophotometer was used to record the ATR-IR spectrum, and recorded in the range of 4000–400 cm^−1^. HRMS measurements were performed by direct infusion to an Orbitrap Exploris 480 (ThermoFisher, Waltham, MA, USA) mass spectrometer equipped with a heated electrospray ion source (HESI).

##### 2-(Benzenesulfonyl)-N-Cyclohexylhydrazine-1-Carbothioamide (WZ1)

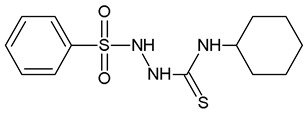



C_13_H_19_N_3_O_2_S_2_ (313.43); yellow solid, yield 60%; m.p. 150–151 °C. IR cm^−1^ 3370 (NH), 2937 (NH), 2853 (NH), 1526 (C=O), 1171 (C=S). ^1^H NMR (600 MHz, DMSO-*d*_6_): δ 1.10–1.64 (m, 10H, 5CH_2_), 3.97 (s, 1H, CH), 7.43 (d, 1H, NH), 7.59 (dd, 2H, CH^3,5^, *J*_3,4_ = 6.2 Hz, *J*_3,5_ = 5.4 Hz), 7.68 (td, 1H, CH^4^, *J* = 8.4), 7.82 (dd, 2H, CH^2,6^, *J*_2,3_ = 5.1 Hz, *J*_2,4_ = 3.9 Hz), 9.37 (s, 1H, NH), 9.87 (s, 1H, NH). ^13^C NMR (300 MHz, DMSO): δ 25.06, 25.51, 31.88, 52.81, 128,32, 128.40, 128.89, 129.63, 129.69, 133.84, 138.22, 180.68. HRMS (*m*/*z*): calculated monoisotopic mass: 314.099143, measured monoisotopic mass: 314.0988.

##### 2-(Benzenesulfonyl)-N-Butylhydrazine-1-Carbothioamide (WZ2)

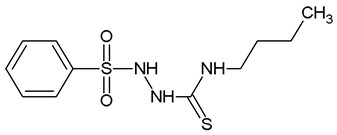



C_11_H_17_N_3_O_2_S_2_ (287.40); yellow solid, yield 85%; m.p. 131–133 °C. IR cm^−1^ 3347 (NH), 3099 (NH), 2956 (NH), 1582 (C=O), 1155 (C=S). ^1^H NMR (600 MHz, DMSO-*d*_6_): δ 1.14–1.16 (m, 3H CH_3_), 1.19–1.44 (m, 4H, 2CH_2_), 3.33–3.41 (m, 2H, CH_2_), 7.58 (dd, 2H, CH^3,5^, *J*_3,4_ = 8.1 Hz, *J*_3,5_ = 3.3 Hz), 7.69 (td, 1H, CH^4^, *J* = 2.1), 7.80 (dd, 2H, CH^2,6^, *J*_2,3_ = 5.1 Hz, *J*_2,4_ = 2.1 Hz), 8.04 (s, 1H, NH), 9.29 (s, 1H, NH), 9.86 (s, 1H, NH). ^13^C NMR (300 MHz, DMSO): δ 14.00, 14.27, 19.81, 31.10, 43.69. 128.04, 128.36, 128.95, 129.47, 129.56, 133.07, 133.75, 138.31, 138.67, 181.92. HRMS (*m*/*z*): calculated monoisotopic mass: 288.083493, measured monoisotopic mass: 288.0832.

##### 2-(Benzenesulfonyl)-N-Hexylhydrazine-1-Carbothioamide (WZ3)

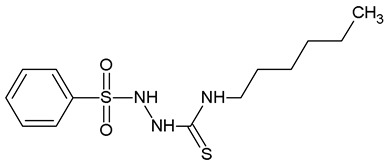



C_13_H_21_N_3_O_2_S_2_ (315.45); yellow solid, yield 80%; m.p. 78–80 °C. IR cm^−1^ 3356 (NH), 3253 (NH), 2916 (NH), 1547 (C=O), 1156 (C=S). ^1^H NMR (600 MHz, DMSO-*d*_6_): δ 0.85–0.88 (t, 3H, CH_3_) 1.20–1.44 (m, 8H, 4CH_2_), 3.37 (t, 2H, CH_2_), 7.59 (dd, 2H, CH^3,5^, *J*_3,4_ = 3.6 Hz, *J*_3,5_ = 1.2 Hz), 7.70 (td, 1H, CH^4^, *J* = 2.1), 7.82 (dd, 2H, CH^2,6^, *J*_2,3_ = 4.8 Hz, *J*_2,4_ = 1.2 Hz),8.04 (s, 1H, NH), 8.41 (s, 1H, NH), 9.29 (s, 1H, NH). ^13^C NMR (300 MHz, DMSO): δ 14.27, 19.88, 31.32, 43.88, 122.93, 127.36, 138.13, 148.93, 149.85, 181.66. HRMS (*m*/*z*): calculated monoisotopic mass: 315.107517, measured monoisotopic mass: 316.1145.

##### 2-(Benzenesulfonyl)-N-Methylhydrazine-1-Carbothioamide (WZ4)

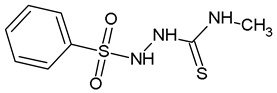



C_8_H_11_N_3_O_2_S_2_ (245.32); yellow solid, yield 92%; m.p. 145–146 °C. IR cm^−1^ 3331 (NH), 3067 (NH), 2996 (NH), 1570 (C=O), 1165 (C=S). ^1^H NMR (600 MHz, DMSO-*d*_6_): δ 2.86 (d, 3H CH_3_), 7.56 (dd, 2H, CH^3,5^, *J*_3,4_ = 3.0 Hz, *J*_3,5_ = 1.2 Hz), 7.69 (td, 1H, CH^4^, *J* = 2.4), 7.82 (dd, 2H, CH^2,6^, *J*_2,3_ = 3.0 Hz, *J*_2,4_ = 2.7 Hz),8.17 (s, 1H, NH), 9.31 (s, 1H, NH), 9.89 (s, 1H, NH). ^13^C NMR (300 MHz, DMSO): δ 31.42, 125.95, 128.93, 130.68, 133.73, 136.63, 142.48, 182.85. HRMS (*m*/*z*): calculated monoisotopic mass: 245.029267, measured monoisotopic mass: 246.0365.

### 4.2. X-ray Structure Determination

X-ray data of WZ1 were collected on the KM4 CCD four-circle diffractometer; crystal sizes 0.60 × 0.30 × 0.20 mm; MoK*α* (*λ* = 0.71973 Å) radiation, *ω* scans, *T* = 293 K, absorption correction: multi-scan [76], *T*_min_/*T*_max_ of 0.463/1.0000. The structures were solved using direct methods using SHELXS2013 [77] and refined by full-matrix least-squares with SHELXL-2014 [78]. The N-bound H atoms were located using difference Fourier synthesis and refined freely. The remaining H atoms were positioned geometrically and treated as riding on their parent C atoms with C–H distances of 0.93 Å (aromatic), 0.97 Å (CH_2_) and 0.940 Å (CH). All H atoms were refined with isotropic displacement parameters taken as 1.5 times those of the respective parent atoms. All calculations were performed using WINGX version 1.64.05 package [79]. CCDC- 2306639 for WZ1 contain the supplementary crystallographic data for this paper. These data can be obtained free of charge at www.ccdc.cam.ac.uk/conts/retrieving.html, (accessed on 8 November 2023) (or from the Cambridge Crystallographic Data Centre (CCDC), 12 Union Road, Cambridge CB2 1EZ, UK; Fax: +44(0) 1223 336 033; email: deposit@ccdc.cam.ac.uk).

Crystal data of WZ1: C_13_H_19_N_3_O_2_S_2_, M = 313.43, monoclinic, space group P2_1_/c, a = 23.864(2), b = 12.472(1), c = 11.0857(7) Å, β = 103.004(8)°, V = 3214.8(5) Å^3^, Z = 8, d_calc_ = 1.295 Mg m^−3^, F(000) = 1328, μ(Mo Kα) = 0.336 mm^−1^, T = 293K, 14219 measured reflections (θ range 1.85–28.58°), 7123 unique reflections, final R = 0.059, wR = 0.142, S = 1.008 for 4000 reflections with I > 2σ(I). 

### 4.3. In Silico Screening and ADMET Predictions

CLC-Pred (Cell Line Cytotoxicity Predictor) 2.0 was used to predict the cytotoxicity of the tested compound [35]. BOILED-Egg was used to predict gastrointestinal absorption [38]. SwissADME service (Swiss Institute of Bioinformatics 2021), a free web tool to evaluate pharmacokinetics, drug-likeness and the medicinal chemistry friendliness of small molecules, was used for an ADME analysis [80]. ProTOX II service was used to predict toxicities for WZ1–WZ4 [39].

### 4.4. Molecular Docking

**Figure 11:**
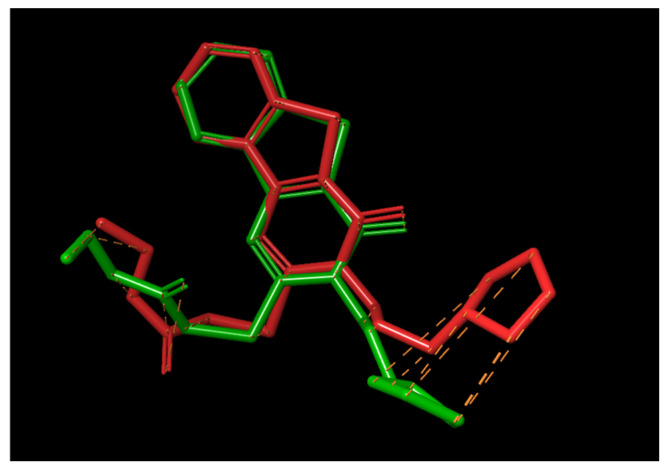
Molecular docking validation (red—redocked ligand, green—native ligand).

The tested compounds (WZ1–WZ4) and the standard human ALDH1A1 inhibitor (PDB code 4X4L [81]) were sketched in the Avogardo 1.2.0 software [82,83] and their geometry was initially optimized using the MMFF94 force field. Ligand, water and cofactors were removed from the protein. Autodock Tools v.1.5.6 software [84] was used for molecular docking. Ligand and protein were prepared according to the method [85], molecular docking was performed using the Lamarckian genetic algorithm [86], where GA runs were set to 50 and the population size was increased to 300; the remaining parameters were considered as default. The tested protein was treated as rigid. A 62 × 108 × 64-point grid box with 0.131 Å spacing was defined by centering the ligand at the active site. The docked ligand poses presented were selected based on the scoring function and visual inspection. To validate the molecular docking parameters, the root mean square deviation (RMSD) of atom pairs of the redocked compound with native ligand was calculated using Maestro 13.4 [87]. Positions with an RMSD close to 2.0 Å were obtained (2.13 Å obtained), which indicated that docking analysis was correct (Figure 11). The Discovery Studio Visualizer [88] generated molecular representations of each protein–ligand complex.

### 4.5. Antimicrobial Activity

#### 4.5.1. Microorganisms

Reference strains of microorganisms that came from American Type Culture Collection (ATCC) (Manassas, VA, USA) were used for this study. The representative Gram-positive bacteria were as follows: *Staphylococcus aureus* ATCC 25923, *Staphylococcus aureus* ATCC 29213, *Staphylococcus aureus* ATCC 43300, *Staphylococcus epidermidis* ATCC 12228, *Enterococcus faecalis* ATCC 29212, *Micrococcus luteus* ATCC 10240, *Bacillus subtilis* ATCC 6633 and *Bacillus cereus* ATCC 10876. While, those of Gram-negative bacteria were as follows: *Bordetella bronchiseptica* ATCC 4617, *Escherichia coli* ATCC 25922, *Klebsiella pneumoniae* ATCC 13883, *Proteus mirabilis* ATCC 12453, *Salmonella* Typhimurium ATCC 14028 and *Pseudomonas aeruginosa* ATCC 9027. Moreover, the following fungi belonging to yeasts were used: *Candida albicans* ATCC 10231, *Candida albicans* ATCC 2091, *Candida parapsilosis* ATCC 22019, *Candida glabrata* ATCC 90030 and *Candida krusei* ATCC 14243. 

#### 4.5.2. In Vitro Antimicrobial Activity Assay

The new compounds WZ1–WZ4 were assayed for both antibacterial and antifungal activity using the broth microdilution method in accordance with the European Committee on Antimicrobial Susceptibility Testing (EUCAST) [89] and Clinical and Laboratory Standards Institute (CLSI) guidelines [90]. The microorganisms were grown at 35 °C for 18–24 h on appropriate media (nutrient agar and Sabouraud for bacteria and fungi, respectively) (BioMaxima S.A., Lublin, Poland). Next, microbial suspensions with an optical density of 0.5 McFarland standard scale (in sterile 0.85% NaCl) were prepared. In turn, the tested compounds were initially diluted at 50 mg/mL in dimethyl sulfoxide (DMSO) and the broth microdilution method was used to assess their minimum inhibitory concentrations (MICs). For these studies, selective broth Mueller-Hinton (MH) (BioMaxima S.A., Lublin, Poland) and RPMI (Roswell Park Memorial Institute) 1640 with MOPS (3-(N-Morpholino)propanesulfonic acid) (Sigma-Aldrich Chemicals, St. Louis, MO, USA) were used for bacteria and fungi, respectively. The antimicrobial effect of compounds WZ1–WZ4 was determined in the concentration range from 2000 to 0.98 µg/mL. 

Then, the microbial suspensions were introduced into each well of the microplate and incubated for 18–24 h at 35 °C. The MIC value (minimum concentration of compounds that showed inhibition of their growth) was determined in the BioTek spectrophotometer (Biokom, Janki, Poland) and compared with control cultures without tested compounds. The reference drugs ciprofloxacin or vancomycin (against bacteria) and nystatin (against fungi) (Sigma-Aldrich Chemicals, St. Louis, MO, USA) were also used. Next, the values of the minimum bactericidal concentration (MBC) and minimum fungicidal concentration (MFC) of WZ1–WZ4 were assessed. For this purpose, the cultures from each MIC determination well were transferred to a suitable solid medium and incubated under appropriate conditions. The lowest concentrations of compounds without microbial growth were read as MBC or MFC. The studies were performed in triplicate and representative data were gathered [89,90,91,92]. 

The interpretation of the obtained results was carried out in accordance with the literature data as follows: no bioactivity (MIC > 1000 µg/mL), mild bioactivity (MIC = 501–1000 µg/mL), moderate bioactivity (MIC = 126–500 µg/mL), good bioactivity (MIC = 26–125 µg/mL), strong bioactivity (MIC = 10–25 µg/mL) and very strong bioactivity (MIC < 10 µg/mL) [91]. In turn, the MBC/MIC or MFC/MIC ratios were used to assess bactericidal/fungicidal (MBC/MIC or MFC/MIC ≤ 4) and bacteriostatic/fungistatic (MBC/MIC or MFC/MIC > 4) effect of these compounds [93]. 

#### 4.5.3. Toxicity to Erythrocyte Assay

The toxicity of WZ1–WZ4 compounds towards red blood cells was tested using the erythrocyte lysis assay (ELA) [61,94,95,96]. Erythrocytes from fresh sheep blood (BioMaxima S.A., Poland) were centrifuged and washed with 0.85% NaCl. Next, 100 μL of a 2% suspension of red blood cells in physiological saline was added to the wells of microtiter plate. The double dilutions of compounds WZ1–WZ4 were prepared in the range of 2000–15.62 μg/mL. Appropriate controls were used to assess the hemolytic potential of tested compounds, i.e., 100% lysis of erythrocytes using 4% Triton X-100 (Pol-Aura, Różnowo, Poland) and 0% lysis in saline solution. Next, all sample plates were incubated (1 h at 37 °C), centrifuged for 10 min to separate unlysed red blood cells, and the supernatant was placed in a new plate. The ELA is a biological test by which the lytic response can be measured spectrophotometrically (at a wavelength of 450 nm) by the amount of hemoglobin released. The hemolysis percentage was calculated according to the following equation:

(1) $$\%\;Hemolysis\;=\;\frac{A450\;of\;test\;compound\;treated\;sample\;-\;A450\;of\;buffer\;treated\;sample}{A450\;of\;4\%\;Triton\;X100\;treated\;samples\;-\;A450\;of\;buffer\;treated\;sample}\;\times \;100$$

### 4.6. Anticancer Activity

#### 4.6.1. Cell Culture

Cancer cell lines originating from different organs, i.e., AGS (stomach—gastric adenocarcinoma), HepG2 (hepatocellular carcinoma), Du145, PC3 (prostate cancer), 769-P (renal cell carcinoma) and physiology cell line WS1 (fibroblasts), were purchased from ATCC. The cell cultures were carried out in an appropriate medium (DMEM or MEM) containing 10% heat-inactivated FBS with penicillin (100 U/mL) and streptomycin (100 U/mL). The cells were kept in a humidified incubator at 37 °C and 5% CO_2_ and passaged three times before experimenting. 

#### 4.6.2. Antiproliferative Activity Assay 

The antiproliferative properties test for each cancer line was performed in 96-well plates, in which cells with a density of 2 × 10^4^ were seeded in 100 µL of medium containing 10% FBS. After 24 h of allowing the cells to attach, the wells were washed with DPBS (Dulbecco’s phosphate-buffered saline) solution and the cells were incubated in fresh medium w/o FBS containing 1 µM, 10 µM, 50 µM, 200 µM or 500 µM of the tested compounds for 24 and 48 h. All substances were first dissolved in DMSO to prepare stock solutions that were then diluted in the medium to obtain a maximum of 0.1% *w*/*v* DMSO in the final solution. Each concentration was tested in triplicate. DMSO at a concentration of 0.1% in the medium was also used as a negative control. The antiproliferative activity of the compounds was tested using the MTT test. After each incubation time, the medium containing the test compounds and the negative control was removed, the cells were washed with DPBS solution, and then 100 µL of FBS-free and antibiotic-free fresh medium containing 0.5 mg/mL MTT was added to each well. The plates were incubated for 3 h in an incubator at 37 °C and 5% CO_2_. After incubation, the medium was removed, the cells were washed with 100 µL of PBS, and 100 µL of DMSO was added to each well to dissolve the purple formazan. The plate was shaken for 3 min and the absorbance was read at 570 nm (BioTek Synergy H1 microplate reader). Cell viability was calculated from the mean of the OD readings. Based on the dose–response curve, IC_50_ values were calculated (MS Excel). From these, therapeutic indices were then calculated for each cell line if the substance exhibited cytostatic properties. Therapeutic index (TI) results were calculated as the ratio of the IC_50_ of physiological cells to the IC_50_ of tumor cells [97].

#### 4.6.3. Determination of Concentration Aldehyde Dehydrogenase 1 Family, Member A1 (ALDH1A1)

Selected cell lines, viz HepG2, Du145 and 769-P, were seeded into 6-well plates and incubated for 24 h after seeding with compounds at a concentration equal to IC_25_. After a further 24 h or 48 h, cells were detached with trypsin and centrifuged. The resulting cell pellet was resuspended in 0.5 mL PBS. The samples were then sonicated, and protein was determined using the Bradford method (Bio-rad; Hercules, CA, USA). Samples were equilibrated to 10 mg/mL of protein, and ALDH1A1 was determined in the samples prepared in this way. ALDH1A1 determination was performed using an ELISA (SEE824Hu; Cloud Clone Corp., Katy, TX, USA), according to the manufacturer’s protocol. 

#### 4.6.4. Statistical Analysis

All analyzed data in *Antiproliferative activity assay* met the normality of distribution. Therefore, they were analyzed by Dunnett’s test. A Kruskal–Wallis ANOVA test was used to analyze the data from the *ALDH1A1 determination*. All data were analyzed using GraphPad Prism 8 software; *p* = 0.05 was taken as the level of statistical significance.

## 5. Conclusions

The X-ray analysis carried out for compound WZ1 shows that this compound crystallizes with two molecules in the asymmetric part of unit cell with very similar geometry and slightly different conformations. These conformations are stabilized by the N–H…N intramolecular hydrogen bond, while the intermolecular hydrogen bonds N–H…S play a key role in crystal packing. Newly synthesized compounds WZ1–WZ4 showed some antimicrobial activity. The activity of substances WZ1, WZ3 and WZ4 was moderate or good against most Gram-positive bacteria (MIC = 62.5–500 µg/mL) and data obtained using tests with erythrocytes confirmed that this antimicrobial effect was observed at their <50% hemolytic activity. All tested compounds reduced the viability of cancer lines with varying selectivity. The WZ2 molecule seems to be the best candidate for further research due to its higher cytotoxic selectivity towards cancer cells. The probable mechanism of action may be related to the effect on ALDH1A1, but further research in this area is required.

## Figures and Tables

**Table 1:** The geometry (Å, °) of the hydrogen bonds in the crystal of WZ1.

D–H…A	D–H	H…A	D…A	D–H…A
N1A–H1A…N5A	0.88 (3)	2.33 (4)	2.672 (3)	104 (3)
N1B–H1B…N5B	0.84 (3)	2.24 (4)	2.681 (3)	113 (3)
N4A–H4A…S3B *^i^*	0.86 (4)	2.49 (4)	3.333 (3)	170 (3)
N4B–H4B…S3A *^i^*	0.87 (4)	2.42 (4)	3.281 (3)	177 (3)
N5A–H5A…S3A *^ii^*	0.95 (4)	2.35 (4)	3.296 (3)	174 (2)
N5B–H5B…S3B *^iii^*	0.85 (3)	2.45 (4)	3.259 (3)	158 (3)

*i* = *x*, *y*, *z*; *ii* = *x*, ½ − *y*, −½ + *z; iii* = *x*, ½ − *y*, ½ + *z.*

**Table 2:** Cancer cell line prediction results for WZ1–WZ4.

Compound	Pa	Pi	Cell-Line	Tissue/Organ
WZ1	0.896	0.005	HepG2	Liver
	0.688	0.008	DU-145	Prostate
WZ2	0.860	0.005	HepG2	Liver
	0.620	0.011	DU-145	Prostate
WZ3	0.857	0.005	HepG2	Liver
	0.607	0.012	DU-145	Prostate
WZ4	0.856	0.005	HepG2	Liver
	0.696	0.008	DU-145	Prostate

Pa (probability “to be active”), Pi (probability “to be inactive”).

**Table 3:** The predicted mechanism of biological activity of **WZ1**–**WZ4**.

Comp.	Pa	Pi	Mechanism of Action
WZ1	0.898	0.005	Aldehyde dehydrogenase 1A1 inhibitor
	0.775	0.034	Lysosomal alpha-glucosidase inhibitor
	0.686	0.003	Threonine aspartase 1 inhibitor
	0.644	0.027	Tyrosyl-DNA phosphodiesterase 1 inhibitor
WZ2	0.856	0.008	Aldehyde dehydrogenase 1A1 inhibitor
	0.720	0.045	Lysosomal alpha-glucosidase inhibitor
	0.657	0.025	Tyrosyl-DNA phosphodiesterase inhibitor
WZ3	0.835	0.010	Aldehyde dehydrogenase 1A1 inhibitor
	0.666	0.056	Lysosomal alpha-glucosidase inhibitor
	0.638	0.028	Tyrosyl-DNA phosphodiesterase 1 inhibitor
WZ4	0.944	0.005	Aldehyde dehydrogenase 1A1 inhibitor
	0.777	0.033	Lysosomal alpha-glucosidase inhibitor
	0.732	0.017	Tyrosyl-DNA phosphodiesterase 1 inhibitor
	0.637	0.046	Pyruvate kinase PKM inhibitor

**Table 4:** Predicted drug–drug interactions via cytochrome P450 (CYP) for WZ1–WZ4.

Potential Inhibition	Compound			
	WZ1	WZ2	WZ3	WZ4
CYP1A2	No	No	No	No
CYP2C19	Yes	No	Yes	No
CYP2C9	No	No	Yes	No
CYP2D6	No	No	No	No
CYP3A4	No	No	No	No

**Table 5:** Binding energy values obtained during docking analysis.

Docked Ligands	ALDH1A1 (PDB 4X4L)	
	Binding Energy (kcal/mol)	Ki (μM)
Native ligand	−8.16	1.04
WZ1	−7.12	6.06
WZ2	−5.77	58.46
WZ3	−6.14	31.82
WZ4	−5.47	98.49

**Table 6:** The activity data of studied compounds WZ1–WZ4 expressed as MIC (MBC or MFC) [µg/mL] and {MBC/MIC or MFC/MIC} values against the reference strains of bacteria and fungi. The standard antimicrobial drugs used as positive controls were ciprofloxacin (CIP) for bacteria (except enterococci), vancomycin (VA*) for enterococci and nystatin (NY**) for fungi.

Species		MIC (MBC or MFC) [µg/mL] and {MBC/MIC or MFC/MIC} Values of the Studied Compounds and Positive Controls				
		WZ1	WZ2	WZ3	WZ4	CIP/VA* /NY**
Gram-positive bacteria	*Staphylococcus* *aureus* ATCC 43300	500 (>2000) {>4}	2000 (>2000) {>1}	250 (500) {2}	500 (2000) {4}	0.24 (0.24) {1}
	*Staphylococcus* *aureus* ATCC 25923	500 (>2000) {>4}	1000 (>2000) {>2}	250 (1000) {4}	500 (>2000) {>4}	0.48 (0.48) {1}
	*Staphylococcus* *aureus* ATCC 29213	500 (>2000) {>4}	2000 (>2000) {>1}	500 (500) {1}	1000 (>2000) {>2}	0.48 (0.48) {1}
	*Staphylococcus* *epidermidis* ATCC 12228	250 (>2000) {>8}	1000 (>2000) {>2}	250 (250) {1}	125 (500) {4}	0.12 (0.12) {1}
	*Enterococcus* *faecalis* ATCC 29212	2000 (>2000) {>1}	>2000 (>2000) {>1}	500 (>2000) {>4}	1000 (>2000) {>2}	0.98 * (1.95) {2}
	*Micrococcus* *luteus* ATCC 10240	500 (>2000) {>4}	1000 (2000) {2}	250 (500) {2}	250 (500) {2}	0.98 (1.95) {2}
	*Bacillus* *subtilis* ATCC 6633	500 (>2000) {>4}	1000 (>2000) {>2}	125 (500) {4}	125 (2000) {16}	0.03 (0.03) {1}
	*Bacillus* *cereus* ATCC 10876	500 (>2000) {>4}	1000 (2000) {2}	250 (250) {1}	62.5 (125) {2}	0.06 (0.12) {2}
Gram-negative bacteria	*Bordetella bronchiseptica* ATCC 4617	>2000 (>2000) {>1}	>2000 (>2000) {>1}	500 (>2000) {>4}	>2000 (>2000) {>1}	0.98 (0.98) {1}
	*Klebsiella* *pneumoniae* ATCC 13883	>2000 (>2000) {>1}	>2000 (>2000) {>1}	1000 (>2000) {>2}	>2000 (>2000) {>1}	0.12 (0.24) {2}
	*Proteus* *mirabilis* ATCC 12453	>2000 (>2000) {>1}	>2000 (>2000) {>1}	1000 (>2000) {>2}	>2000 (>2000) {>1}	0.03 (0.03) {1)
	*Salmonella* Typhimurium ATCC 14028	>2000 (>2000) {>1}	>2000 (>2000) {>1}	1000 (>2000) {>2}	>2000 (>2000) {>1}	0.06 (0.06) {1}
	*Escherichia* *coli* ATCC 25922	>2000 (>2000) {>1}	>2000 (>2000) {>1}	500 (>2000) {>4}	>2000 (>2000) {>1}	0.004 (0.008) {2}
	*Pseudomonas aeruginosa* ATCC 9027	>2000 (>2000) {>1}	>2000 (>2000) {>1}	1000 (1000) {1}	>2000 (>2000) {>1}	0.48 (0.98) {2}
Fungi	*Candida* *albicans* ATCC 2091	>2000 (>2000) {>1}	2000 (>2000) {>1}	500 (1000) {2}	250 (1000) {4}	0.24 ** (0.24) {1}
	*Candida* *albicans* ATCC 10231	>2000 (>2000) {>1}	2000 (>2000) {>1}	500 (1000) {2}	500 (1000) {2}	0.48 ** (0.48) {1}
	*Candida* *parapsilosis* ATCC 22019	>2000 (>2000) {>1}	2000 (>2000) {>1}	2000 (2000) {1}	125 (1000) {8}	0.24 ** (0.48) {2}
	*Candida* *glabrata* ATCC 90030	>2000 (>2000) {>1}	>2000 (>2000) {>1}	2000 (2000) {1}	125 (500) {4}	0.24 ** (0.48) {2}
	*Candida* *krusei* ATCC 14243	>2000 (>2000) {>1}	2000 (>2000) {>1}	500 (1000) {2}	500 (2000) {4}	0.24 ** (0.24) {1}

## Data Availability

Data is contained within the article and supplementary material.

## Supplementary Materials

The following supporting information can be downloaded at: https://www.mdpi.com/article/10.3390/ph16121706/s1, Figure S1. NMR spectra for WZ1, Figure S2. NMR spectra for WZ2, Figure S3. NMR spectra for WZ3, Figure S4. NMR spectra for WZ4, Figure S5. MS spectra for compounds WZ1–WZ4.
