# Supplementary material for: N-Substituted 2-(Benzenosulfonyl)-1-Carbotioamide Derivatives Exert Antimicrobial and Cytotoxic Effects via Aldehyde Dehydrogenase Pathway: Synthesis, In Silico and In Vitro Studies
Source: Pharmaceuticals (Basel). 2023 Dec 8;16(12):1706. doi: 10.3390/ph16121706 (PMC10747733; doi:10.3390/ph16121706)

SUPLEMENTARY MATERIAL

N-Substituted 2-(benzenosulfonyl)-1-carbotioamide derivatives exert antimicrobial and cytotoxic effects via aldehyde dehydrogenase pathway; synthesis, *in silico* and *in vitro* studies

Lucja Justyna Walczak-Nowicka [1], Anna Biernasiuk [2], Wojciech Ziemichód [3], Zbigniew Karczmarzyk [4], Mateusz Kwaśnik [5], Paweł Kozyra [3], Waldemar Wysocki [4], Agnieszka Stenzel-Bembenek [6], Dorota Kowalczuk [7], Mariola Herbet [1] and Monika Pitucha [3,*]

[1] Chair and Department of Toxicology, Faculty of Pharmacy, Medical University of Lublin, Jaczewskiego 8b, 20-090 Lublin, Poland
[2] Department of Pharmaceutical Microbiology, Faculty of Pharmacy, Medical University of Lublin, Chodzki 1, 20-093 Lublin, Poland
[3] Independent Radiopharmacy Unit, Faculty of Pharmacy, Medical University of Lublin, Chodzki 4a, 20-093 Lublin, Poland
[4] Institute of Chemistry, University of Siedlce, 3 Maja 54, 08-110 Siedlce, Poland
[5] Department of Molecular Biology, Faculty of Medicine, The John Paul II Catholic University of Lublin, Konstantynów 1J/4.03, 20-708 Lublin, Poland
[6] Department of Biochemistry and Molecular Biology, Faculty of Medical Sciences, Medical University of Lublin, Chodzki 1, 20-093 Lublin, Poland
[7] Department of Medicinal Chemistry, Faculty of Pharmacy, Medical University of Lublin, Jaczewskiego 4, 20-090, Lublin, PL, Poland
* Correspondence: monika.pitucha@umlub.pl (MP);

Figure S1. NMR spectra for WZ1

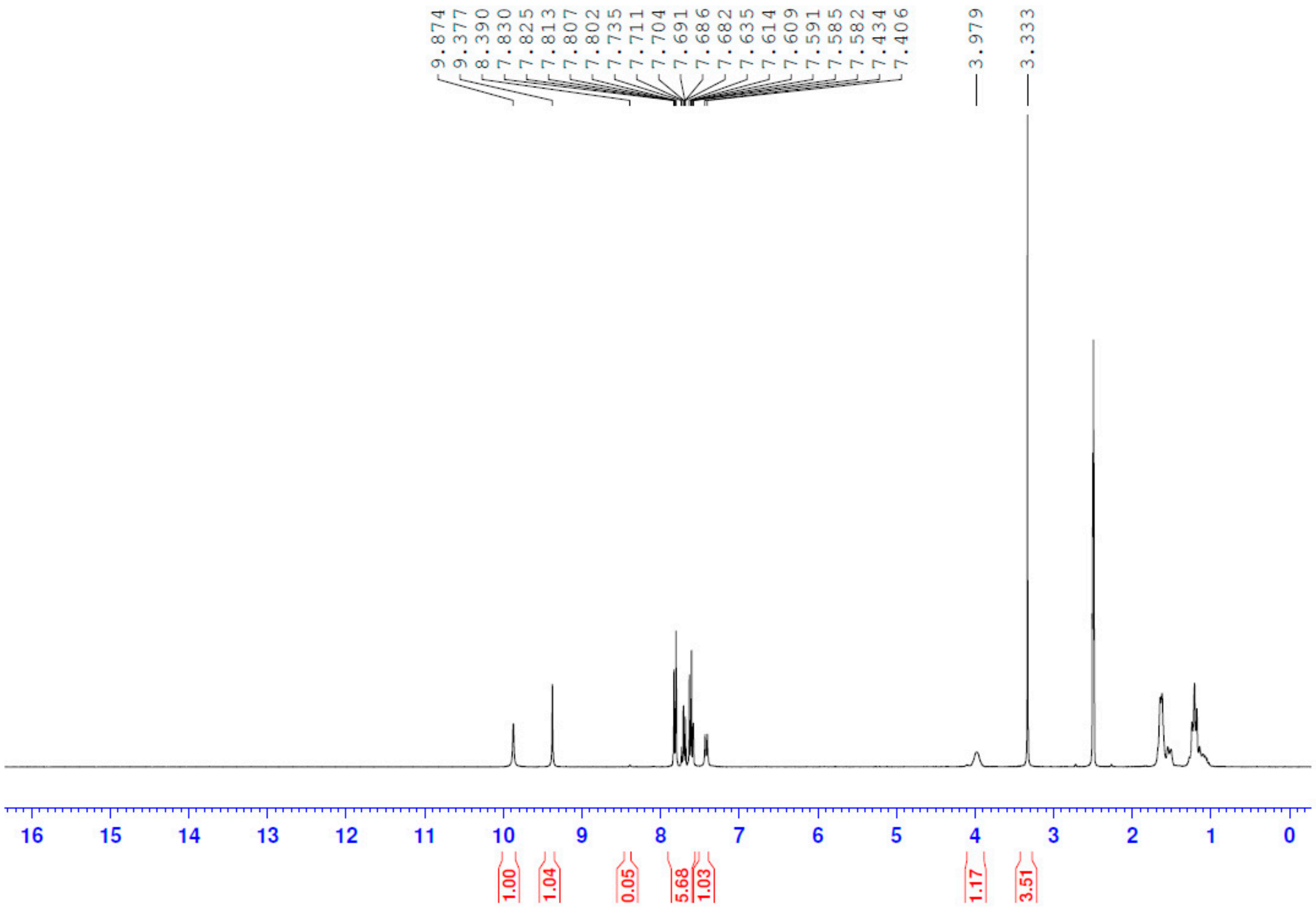

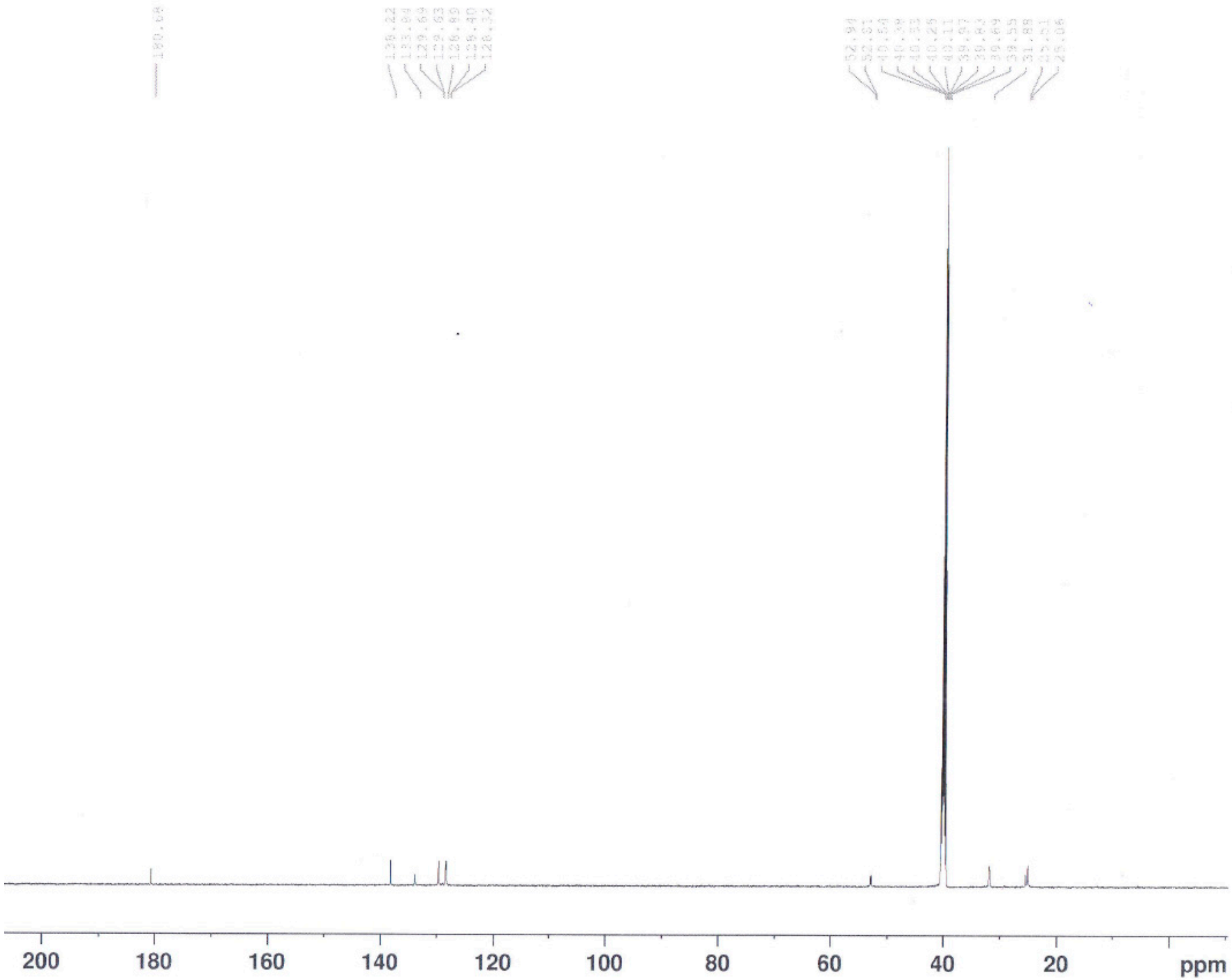

180.68
138.22
133.84
129.69
129.63
128.89
128.40
128.32
52.94
52.81
40.54
40.39
40.33
40.25
40.11
39.97
39.83
39.69
39.55
31.88
25.21
25.06
200
180
160
140
120
100
80
60
40
20
ppm

Figure S2. NMR spectra for WZ-2

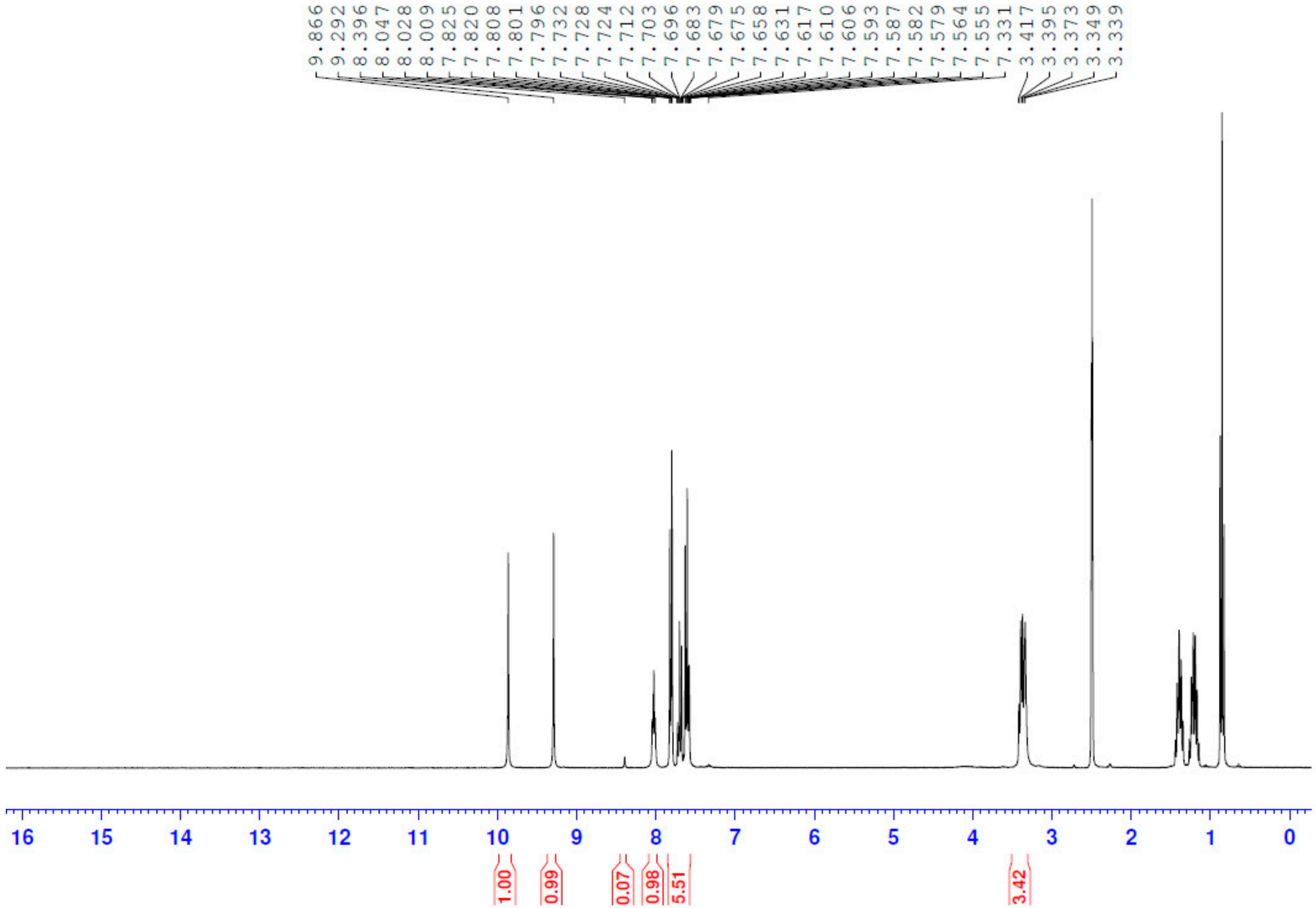

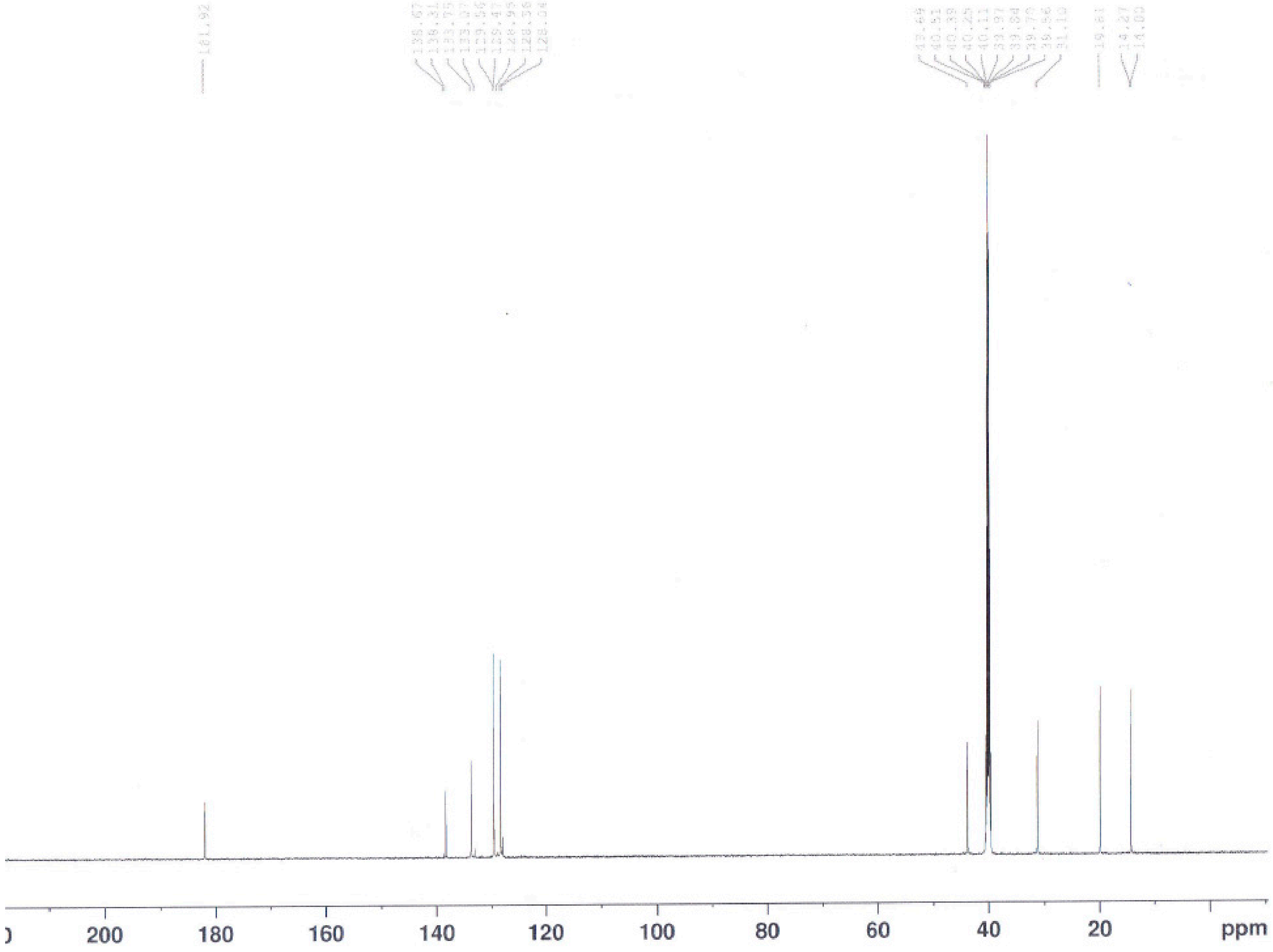

181.92
138.67
138.31
133.75
133.37
129.56
129.47
128.95
128.38
128.04
43.69
40.53
40.39
40.25
40.11
39.97
39.84
39.70
39.56
31.10
19.81
14.27
14.03
200
180
160
140
120
100
80
60
40
20
ppm

Figure S3. NMR spectra for WZ-3

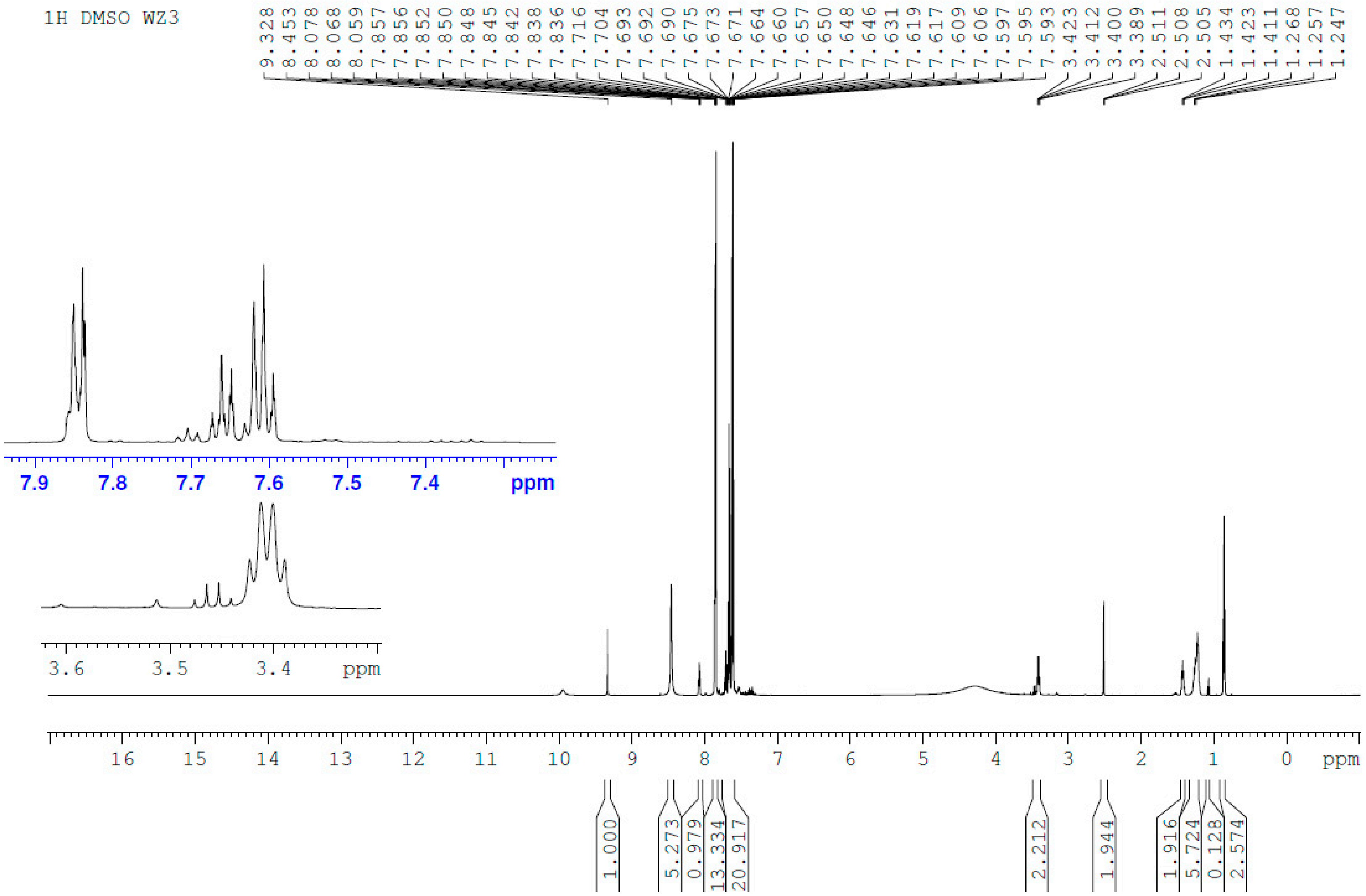

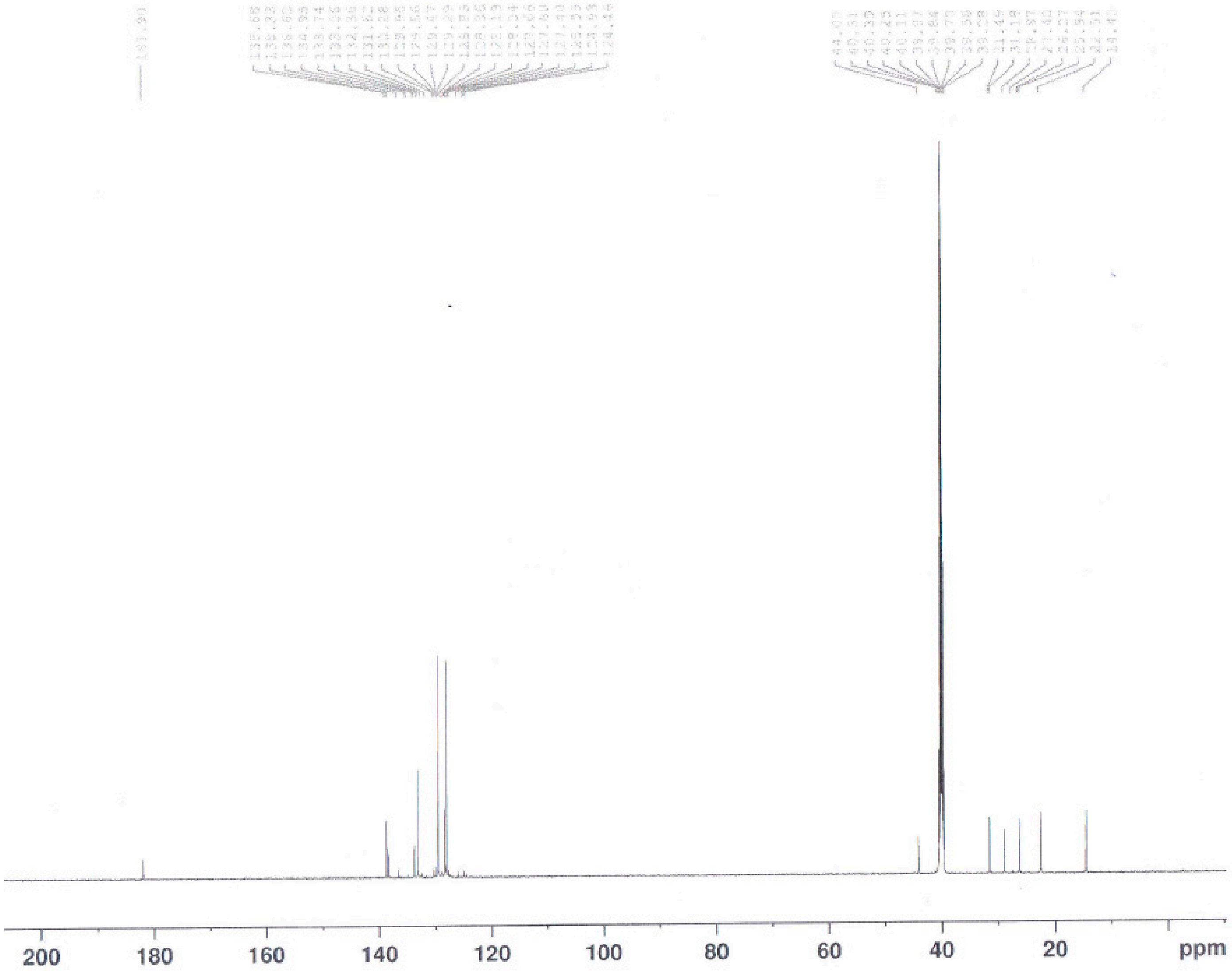

200
180
160
140
120
100
80
60
40
20
ppm

Figure S4. NMR spectra for WZ-4

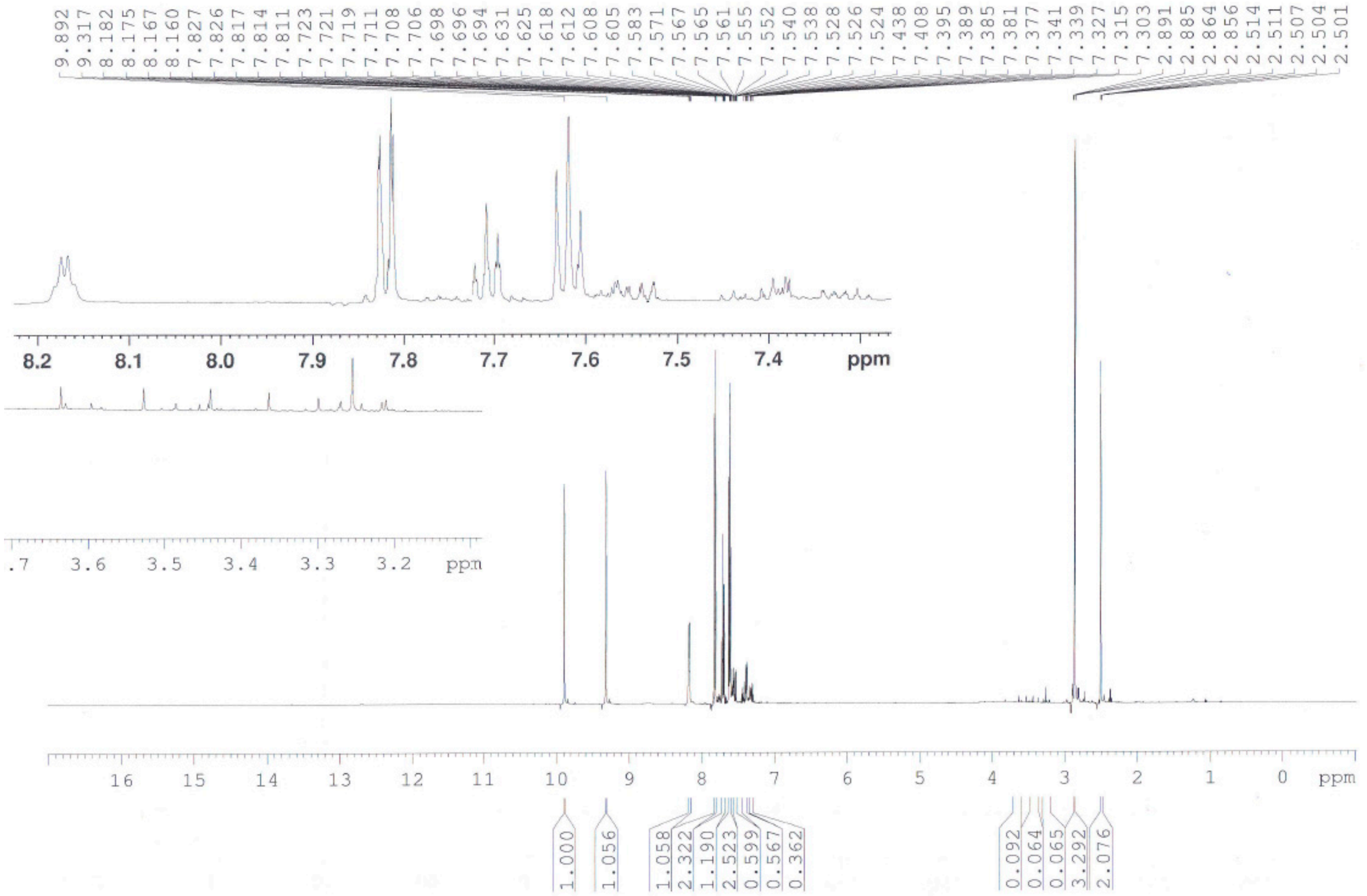

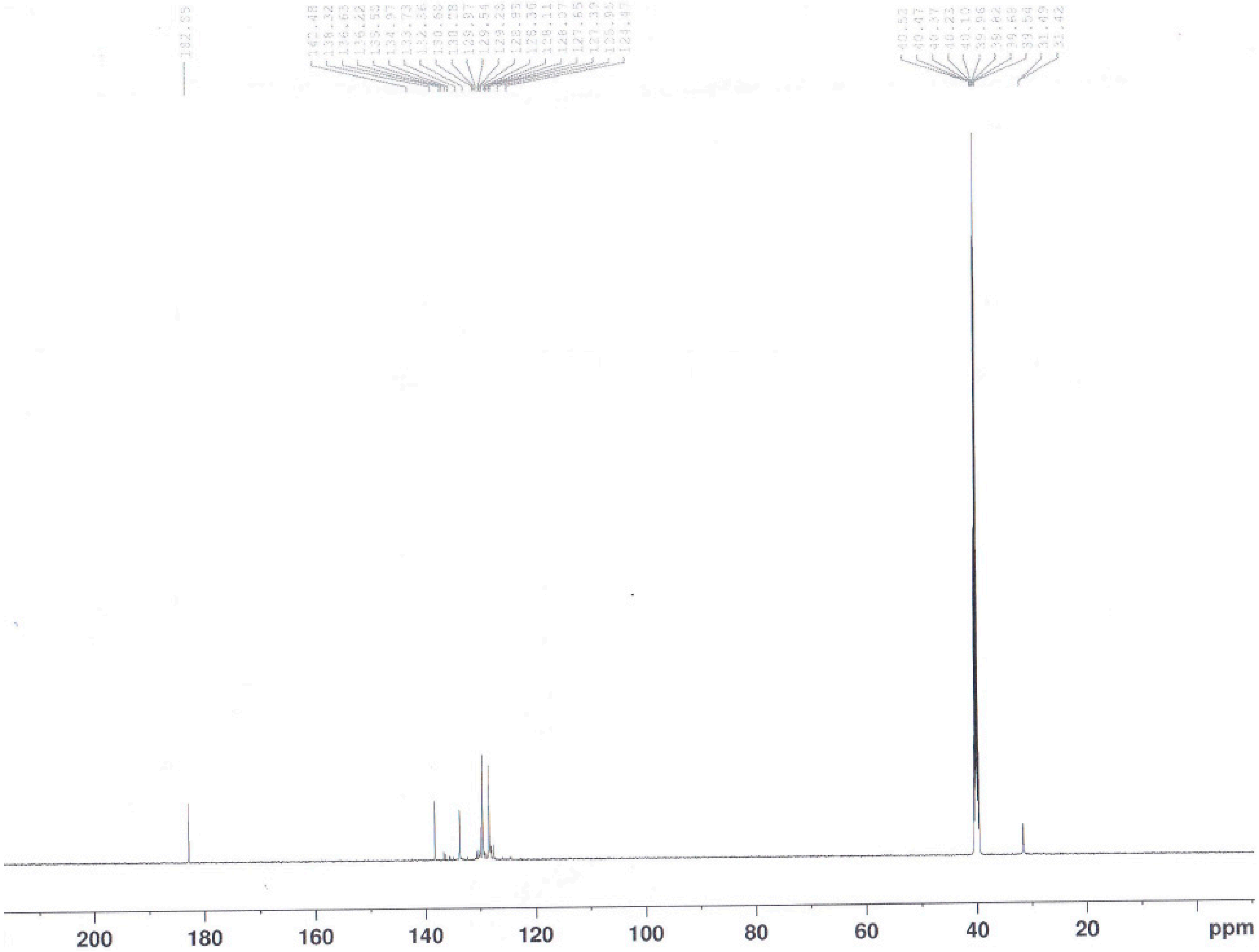
182.85
142.48
138.32
136.63
136.22
135.55
134.97
133.73
132.36
130.68
130.28
129.87
129.54
129.28
128.95
128.36
128.11
128.07
127.55
127.39
125.95
124.47
40.53
40.47
40.37
40.23
40.10
39.96
39.82
39.68
39.54
31.49
31.42
200
180
160
140
120
100
80
60
40
20
ppm

Figure S5. MS spectra for compounds WZ1-WZ-4

	Measured HRMS spectrum (compound isotopic pattern)
WZ-1	

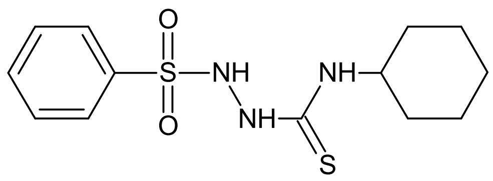

Molecular Formula: $C_{13}H_{19}N_3O_2S_2$
Monoisotopic Mass: 313.091867 Da
[M+H]+: 314.099143 Da

measured mass (Da): 314.0988
mass difference (ppm): 1.04

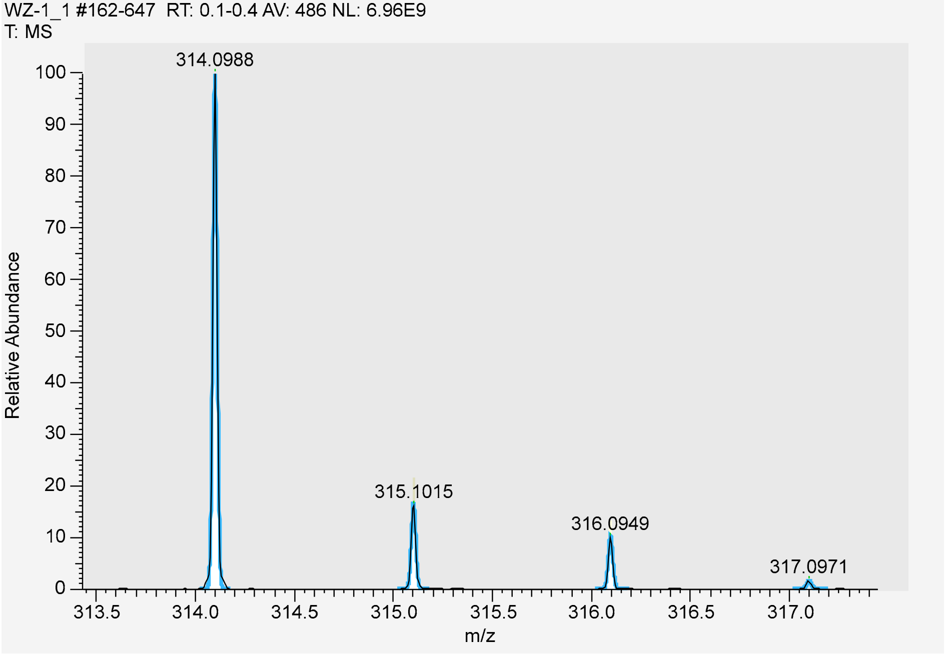

WZ-2

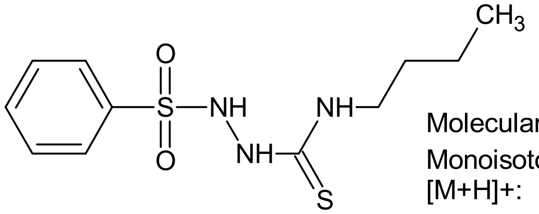

Molecular Formula: $C_{11}H_{17}N_3O_2S_2$
Monoisotopic Mass: 287.076217 Da
[M+H]+: 288.083493 Da

measured mass (Da): 288.0832
mass difference (ppm): 1.15

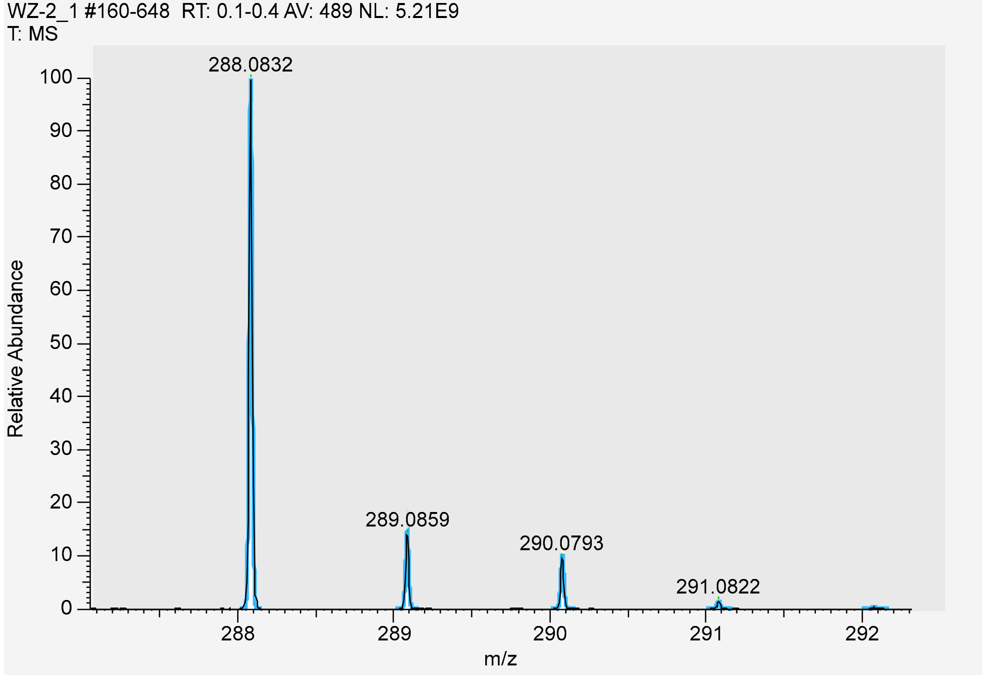

WZ-3

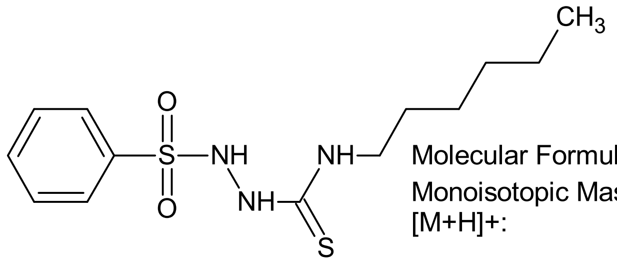

Molecular Formula: $C_{13}H_{21}N_3O_2S_2$
Monoisotopic Mass: 315.107517 Da
[M+H]+: 316.114793 Da

measured mass (Da): 316.1145
mass difference (ppm): 0.87

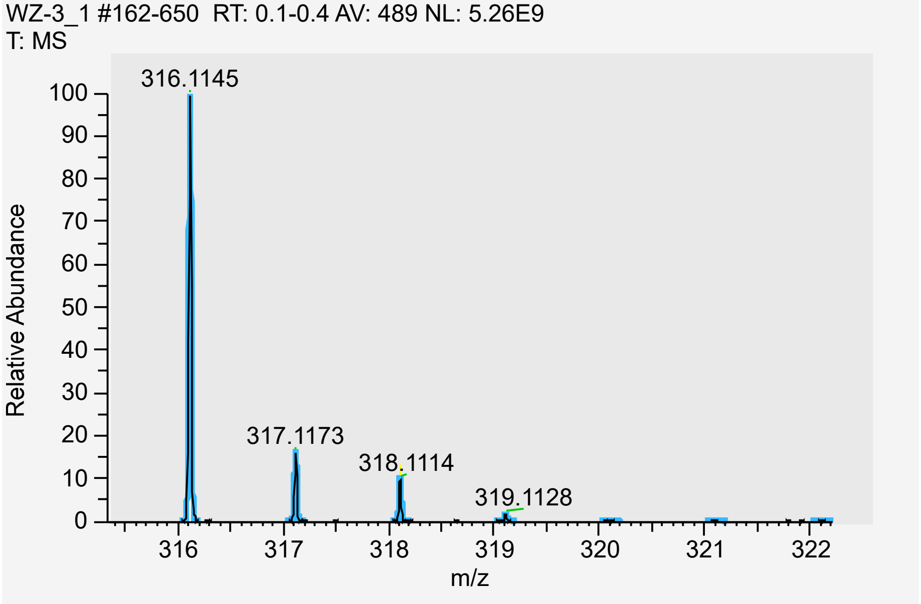

WZ-4

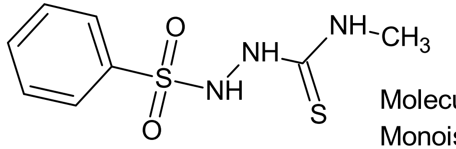

Molecular Formula: $C_8H_{11}N_3O_2S_2$
Monoisotopic Mass: 245.029267 Da
[M+H]+: 246.036543 Da

measured mass (Da): 246.0365
mass difference (ppm): 0.01

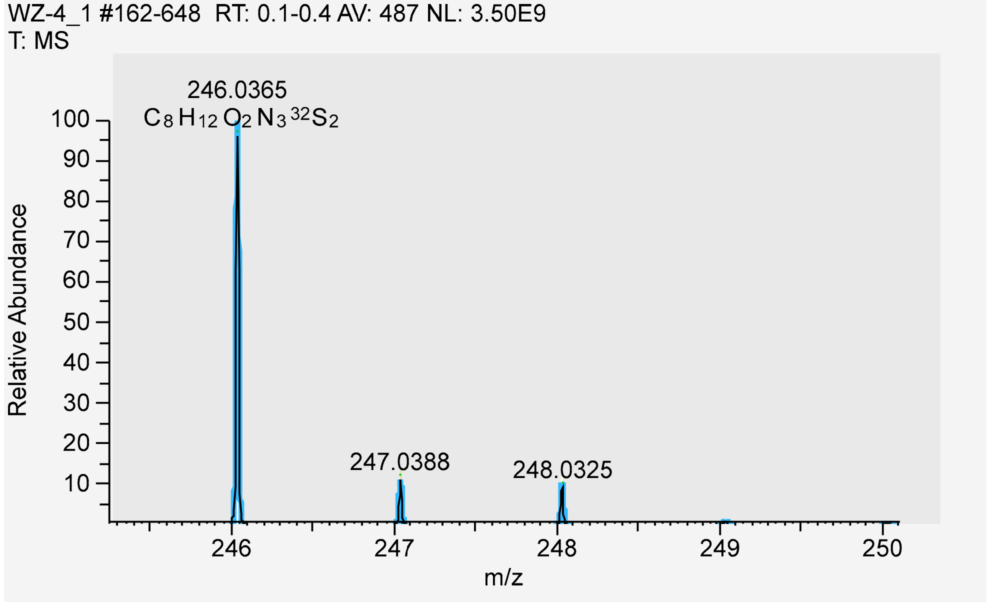

Supplement: Supplementary file 1 [file pharmaceuticals-16-01706-s001.zip › pharmaceuticals-2737172-supplementary.pdf]
